# Conservation and Variability of West Nile Virus Proteins

**DOI:** 10.1371/journal.pone.0005352

**Published:** 2009-04-29

**Authors:** Qi Ying Koo, Asif M. Khan, Keun-Ok Jung, Shweta Ramdas, Olivo Miotto, Tin Wee Tan, Vladimir Brusic, Jerome Salmon, J. Thomas August

**Affiliations:** 1 Department of Biochemistry, Yong Loo Lin School of Medicine, National University of Singapore, Singapore, Singapore; 2 Department of Pharmacology and Molecular Sciences, The Johns Hopkins University School of Medicine, Baltimore, Maryland, United States of America; 3 MRC Centre for Genomics and Global Health, University of Oxford, Oxford, United Kingdom; 4 Mahidol-Oxford Research Unit, Faculty of Tropical Medicine, Mahidol University, Bangkok, Thailand; 5 Cancer Vaccine Center, Dana-Farber Cancer Institute, Boston, Massachusetts, United States of America; Institute of Infectious Disease and Molecular Medicine, South Africa

## Abstract

West Nile virus (WNV) has emerged globally as an increasingly important pathogen for humans and domestic animals. Studies of the evolutionary diversity of the virus over its known history will help to elucidate conserved sites, and characterize their correspondence to other pathogens and their relevance to the immune system. We describe a large-scale analysis of the entire WNV proteome, aimed at identifying and characterizing evolutionarily conserved amino acid sequences. This study, which used 2,746 WNV protein sequences collected from the NCBI GenPept database, focused on analysis of peptides of length 9 amino acids or more, which are immunologically relevant as potential T-cell epitopes. Entropy-based analysis of the diversity of WNV sequences, revealed the presence of numerous evolutionarily stable nonamer positions across the proteome (entropy value of ≤1). The representation (frequency) of nonamers variant to the predominant peptide at these stable positions was, generally, low (≤10% of the WNV sequences analyzed). Eighty-eight fragments of length 9–29 amino acids, representing ∼34% of the WNV polyprotein length, were identified to be identical and evolutionarily stable in all analyzed WNV sequences. Of the 88 completely conserved sequences, 67 are also present in other flaviviruses, and several have been associated with the functional and structural properties of viral proteins. Immunoinformatic analysis revealed that the majority (78/88) of conserved sequences are potentially immunogenic, while 44 contained experimentally confirmed human T-cell epitopes. This study identified a comprehensive catalogue of completely conserved WNV sequences, many of which are shared by other flaviviruses, and majority are potential epitopes. The complete conservation of these immunologically relevant sequences through the entire recorded WNV history suggests they will be valuable as components of peptide-specific vaccines or other therapeutic applications, for sequence-specific diagnosis of a wide-range of *Flavivivirus* infections, and for studies of homologous sequences among other flaviviruses.

## Introduction

West Nile virus (WNV) is a mosquito-borne pathogen of the family *Flaviviridae*, genus *Flavivi*rus, closely related to other important human pathogens, such as yellow fever (YFV), Japanese encephalitis (JEV), and dengue (DENV) viruses, among others. The genome is a single-stranded positive-sense RNA encoding a polyprotein precursor of approximately 3,430 amino acids, which is cleaved into three structural (capsid, C; precursor membrane and membrane, prM/M; envelope, E) and seven nonstructural proteins (NS1, 2a, 2b, 3, 4a, 4b and 5) [1], [2]. WNV is present predominantly amongst avian hosts, and can infect humans through incidental zoonotic transmission via mosquitoes [3]. The virus is endemic in many parts of Africa, Asia, Europe, and most recently North America [4]. At present, there is no registered human vaccine or specific therapy to prevent or treat WNV infection. Although the majority of infected humans remain asymptomatic, about 20% experience influenza-like symptoms, and approximately 1 in 150 develop severe illness, including meningoencephalitis [5], [6].

Like other RNA viruses, WNV exhibits significant genetic diversity, a consequence of mainly high mutation rates in RNA replication, and subsequent selection of mutants adapted to changing environment [7], [8]. Five distinct genotypes have been identified by phylogenetic analyses of the C-prM-E region, differing from each other by 20–25% across the complete genome [9]. However, variability is uneven across the viral genome, since mutations detrimental to viral fitness are restricted. Thus, while certain protein sites permit multiple mutations, sites critical to viral structure-function are evolutionarily robust and highly conserved. The analysis of the evolutionary dynamics and immunogenic properties of these sites has relevance to multiple applications, including the design of diagnosis, drugs and vaccines.

The focus of this study is to identify and characterize WNV protein regions that have exhibited strong conservation throughout the recorded history of the virus, and that are potential targets of T-cell immune responses. T-cell responses have been implicated in the control and clearance of WNV infection [10]–[15]. Their specificities are governed by human leukocyte antigen (HLA) binding to peptides, derived from proteolysis of antigen (Ag) proteins, for presentation to T cells. HLA class I and class II molecules present peptides to CD8^+^ and CD4^+^ T-cells respectively, which play a critical role in cytotoxic responses and in the induction and maintenance of Ag-specific memory responses.

A bioinformatics approach is applied herein to (a) examine the large number of WNV sequences available in public databases, (b) analyze the conservation and variability of these sequences, (c) identify sequence fragments of WNV proteins that are completely conserved in all known WNVs (henceforth referred to as pan-WNV sequences), (d) examine the structure-function relationship and distribution in nature of pan-WNV sequences, and (e) assess the immune relevance of pan-WNV sequences as potential T-cell epitopes, correlating immunoinformatic predictions to previously reported human WNV T-cell epitopes and to our current studies in the identification of human WNV T-cell epitopes by use of HLA transgenic mice.

## Methods

### Methodology overview

**Figure 1:**
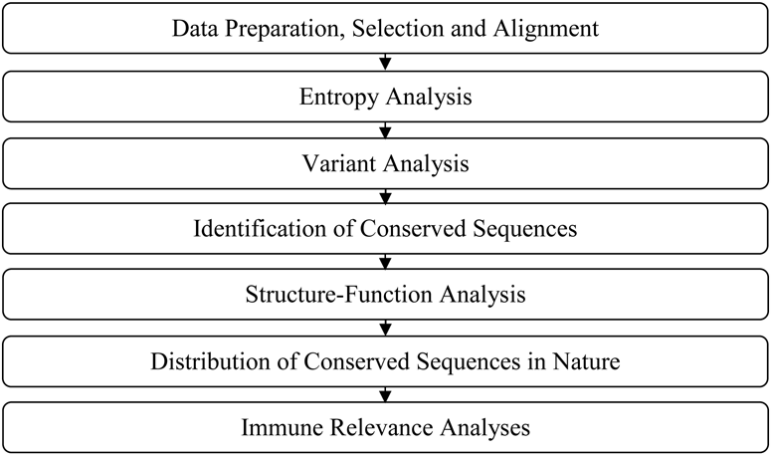
General overview of the bioinformatics approach employed in this study.

The overall bioinformatics approach to this study is summarized in **Figure 1**. The rationale for this approach and methodology is consistent with that of previous studies [16]–[18], and is briefly described below.

### Data preparation, selection and alignment

WNV sequence records were retrieved from the NCBI Entrez protein database [19] in June 2007 by searching the NCBI taxonomy browser for WNV (taxonomy ID 11082).

The sequences of the 10 WNV proteins (C, prM, E, NS1, NS2a, NS2b, NS3, NS4a, NS4b and NS5) were extracted from the downloaded records by performing BLAST [20] against the record sequences using the individual protein sequences as queries, obtained from the annotated WNV reference record P06935 (Swiss-Prot/TrEMBL [21]). Multiple sequence alignment of extracted sequences for each viral protein was performed by use of MUSCLE v3.6 [22]. All multiple sequence alignments were manually inspected and corrected wherever necessary. All extracted WNV protein sequences, whether partial or full-length, were included in all analyses, unless otherwise indicated in the sections below.

In large-scale proteomic analyses such as this study, bias may result from the collection of redundant sequences, derived from identical or highly similar WNV isolates sequenced by surveillance programs. We retained the duplicate sequences (2,206) for our analysis because they reflect the incidence of the corresponding WNV isolates in nature and, further, they do not affect the identification of WNV sequences that are completely (100%) conserved. As for the highly similar sequences, which may have been generated from large sequencing projects during single outbreaks; their removal was deemed undesirable, since such arbitrary selection would introduce additional bias.

### Amino acid difference between WNV protein sequences

Pair-wise percentage amino acid difference for the full-length unique sequences of each WNV proteins was computed by use of ClustalW 1.83 [23] with default parameters. This was done to survey the extent of amino acid variation in the WNV data of 2007.

### Nonamer entropy analysis of WNV sequences

Entropy analysis [24], [25] was carried out as described in [17], by use of the Antigenic Variability Analyser tool (AVANA), to study the diversity of WNV protein sequences over the period which the sequences were collected. Entropy measurements were based on 9 amino acid peptides (nonamers), since it is the typical length of epitopes that bind to HLA class I molecules, and of the binding cores of HLA class II epitopes [26]. At any given position *x* in the alignment, nonamer peptide entropy *H(x)* was calculated by
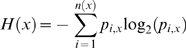
where *p_i,x_* represents the measured probability of a particular nonamer peptide *i* occurring with its center position at *x*; *n(x)* represents the total number of peptides observed at position *x* – larger number of peptides generally result in increased entropy values. Positions with highly dominant (conserved) peptides are characterized by low entropy values, approaching zero. In the case of incomplete sequences, only sequences with a valid nonamer centered at position *x* were included in the computation, and positions where more than 50% of sequences contained gaps were discarded for their low statistical significance. Since peptide entropy is computed at a nonamer's center position, the first and last four positions in each protein alignment are not assigned peptide entropy values. For sequence sets of finite size, entropy calculations are affected by size bias, which were corrected by a statistical sub-sampling method, as described in [17].

### Nonamer variant analysis of WNV sequences

Variable amino acid sites were further analyzed by computing the representation of nonamer variants. At any given position *x*, variant nonamers were defined as nonamers that differed by at least one amino acid from the predominant nonamer (the most common peptide) at that position. Further details of this analysis are given in [17].

### Identification of completely conserved WNV sequences

Completely conserved sequences (pan-WNV sequences) of at least 9 amino acids and fully identical in all the sequences analyzed (100% representation) were identified from the multiple sequence alignment of each protein. Peptides containing unknown residues (X) were ignored.

### Structure-function analysis of pan-WNV sequences

A literature search was conducted to identify reported and putative functional properties of the pan-WNV sequences, including a search of the Prosite database [27] using ScanProsite [28], and a search of the Pfam database [29]. The conserved sequences were also mapped onto the three-dimensional (3-D) structures of WNV proteins whenever these were available in the protein data bank (PDB) [30]. Only 3-D structures obtained via X-ray diffraction were utilized for mapping, and were visualized by use of the CPK representation in the ICM Browser v3.5 (www.molsoft.com).

### Identification of pan-WNV sequences common to other viruses and organisms

Pan-WNV sequences with at least 9 consecutive amino acids in common with other viruses were identified by performing BLAST search against protein sequences of all viruses (txid10239) reported at NCBI (as of August 2007), except WNV (txid11082): parameters included search by Entrez query limited to “txid10239[Organism:exp] NOT txid11082[Organism:exp]”, “automatically adjust parameters for short sequences” option disabled, “low-complexity” filter disabled, maximum number of aligned sequences to be displayed set to “20,000”, expect threshold set to “200,000”, or “20,000”, or “2,000” until a valid result was obtained, word size set to “2”, matrix set to “PAM30”, gap costs set to “Existence: 9, Extension: 1”, compositional adjustments set to “no adjustment”. Similar BLAST searches were carried out against protein sequences of all organisms, except viruses: parameters were the same as the previous search against all viruses, excluding WNV, except that the search by Entrez query was limited to “Root[ORGN] NOT Viruses[ORGN] NOT txid81077[ORGN]”. Artificial sequence hits were removed by the “NOT txid81077[ORGN]” keyword.

### Identification of known and predicted WNV HLA-supertype binding epitopes

A literature search and a search of the Immune Epitope Database [31] (www.immuneepitope.org) identified previously reported HLA class I and II human T-cell epitopes of WNV that overlapped at least 9 consecutive amino acids of pan-WNV sequences. In addition, four prediction models were used to identify candidate WNV sequences that bind to multiple HLA class I or II supertype alleles. Putative HLA class I supertype-restricted peptides were predicted by use of NetCTL [32], Multipred [33], ARB [34], and class II supertype-restricted peptides by Multipred and TEPITOPE [35], following the specifications as described in [17].

## Results

### WNV protein sequence datasets

A total of 2,746 complete and partial WNV protein sequences were extracted from the NCBI Entrez protein database records as of June 2007 (**Table 1** **; Data Set S1**). The large number of sequences analyzed and their wide spatial and temporal (1955–2005; based on information available in annotated NCBI records) distribution enabled a broad survey of WNV protein diversity in nature. The distribution of these sequences varied considerably among the different proteins (from 141 NS4b sequences to 927 E sequences). Comparisons of amino acid variation between the full-length unique sequences of the 10 WNV proteins showed that C had the highest range of amino acid differences across the sequences (up to 23%), while NS4b had the lowest (up to 8%) (**Table 1**).

**Table 1:** Number of WNV protein sequences retrieved from NCBI and their maximum percentage amino-acid difference.

WNV protein	Total length (aa) b	No. of sequences analysed a	% maximum amino-acid difference c
C	123	264	23
prM	167	417	19
E	497	927	12
NS1	352	164	16
NS2a	231	143	20
NS2b	131	146	10
NS3	619	146	10
NS4a	149	142	14
NS4b	256	141	8
NS5	905	256	10
*Total*	*3430*	*2746*	*-*

a Retrieved from NCBI Entrez Protein Database on 28^th^ June 2007.

b Approximate size indicated in number of amino acids.

c Maximum percentage amino-acid difference for each WNV protein, computed using ClustalW [23].

### Evolutionary stability of WNV

**Figure 2:**
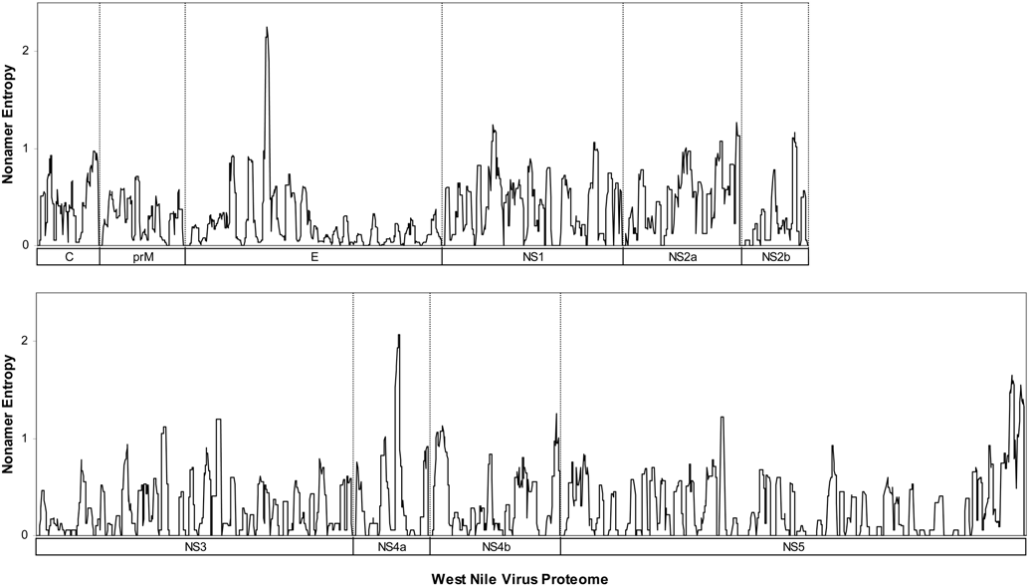
Peptide entropy plots for WNV protein alignments.

The evolutionary diversity of WNV was studied by computing entropy as described in the Methods. The entropy plot revealed the evolutionary variability of nonamer sequences across the WNV proteome (**Figure 2**). The vast majority of nonamer positions exhibited low to moderate entropy (≤1.0), indicating lower probability of mutations occurring over time. Many regions had zero entropy, signifying no change throughout the recorded history of the virus. Peak or near peak entropy values (∼2) were observed in the E, NS4a and NS5 proteins. The NS5 protein, known to be one of the most conserved across *Flavivirus* proteins, had the highest percentage of completely conserved nonamer regions, but also exhibited high entropy in regions at the C-terminal of the protein. Overall, entropy analysis revealed numerous highly conserved and evolutionarily stable WNV sequences distributed throughout the viral proteins, indicative of high genetic stability of WNV, despite its adaptability to global emergence.

### Representation of variant WNV sequences

**Figure 3:**
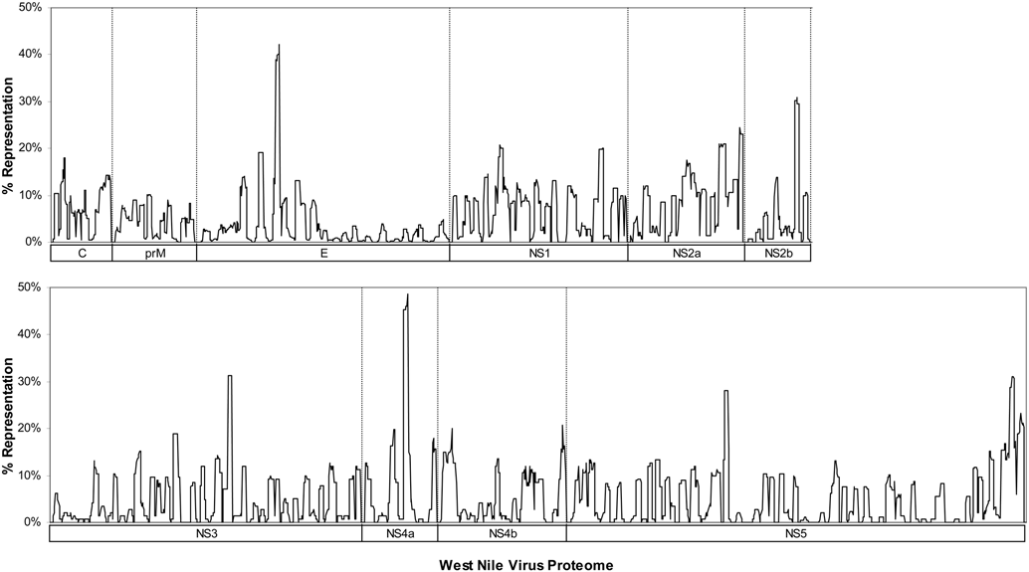
Percentage representation of nonamer variants in relation to the predominant nonamer peptide for all nonamer positions in WNV protein alignments.

Completely conserved nonamer sites with zero variant were numerous and the occurrence of variant nonamer sequences across the WNV proteome was generally low, less than 10% of all WNV recorded sequences at most positions (**Figure 3**). The position with the highest representation of variant nonamer sequences (49%) was found in the nonstructural protein NS4a. Overall, our data suggest a low probability of immune challenges from variant WNV T-cell epitopes, due to a high representation of historically conserved sequences of the WNV proteome in the known virus data.

### Completely conserved pan-WNV sequences

A total of 88 completely conserved sequence fragments (pan-WNV sequences) were identified across the whole proteome (**Table 2**). The length of these fragments ranged from 9 to 29 amino acids, covering a total length of 1,169 amino acids (∼34%) of the complete WNV polyprotein (3,430 aa) (**Table 3**). The C protein had no completely conserved nonamer fragment, which is consistent with the large number of amino acid difference (23%) observed for sequences of this protein, compared to other WNV proteins (**Table 1**). The NS3 and NS5 proteins contained the greatest number of completely conserved fragments, 25 in NS3 (spanning 48% of the protein length) and 30 in NS5 (spanning 51% of the protein length). The other nonstructural proteins NS1, NS2a, NS2b, NS4a and NS4b collectively contained a total of 24 completely conserved sequences, covering 11% to 40% of their respective protein lengths. In contrast, the variability of the structural proteins was much greater: prM had only two completely conserved sequences (14% of the protein length), while E had 7 (18% of the protein length).

**Table 2:** Completely conserved sequence fragments (pan-WNV sequences) of WNV proteins.

WNV protein	Length (aa)	Pan-WNV sequence a
C	-	None
prM	14	125-ESWILRNPGYALVA-138
	10	158-LLLLVAPAYS-167
E	11	1-FNCLGMSNRDF-11
	14	104-GCGLFGKGSIDTCA-117
	9	293-LKGTTYGVC-301
	19	338-SVASLNDLTPVGRLVTVNP-356
	12	370-ELEPPFGDSYIV-381
	10	417-LGDTAWDFGS-426
	12	449-LFGGMSWITQGL-460
NS1	11	58-RSVSRLEHQMW-68
	9	114-GWKAWGKSI-122
	9	154-EVEDFGFGL-162
	10	195-HSDLSYWIES-204
	25	209-TWKLERAVLGEVKSCTWPETHTLWG-233
	11	276-DFDYCPGTTVT-286
	10	313-CRSCTLPPLR-322
	10	328-GCWYGMEIRP-337
NS2a	10	4-DMIDPFQLGL-13
	15	69-NSGGDVVHLALMATF-83
NS2b	10	1-GWPATEVMTA-10
	14	12-GLMFAIVGGLAELD-25
	9	32-PMTIAGLMF-40
	11	108-SAYTPWAILPS-118
NS3	10	1-GGVLWDTPSP-10
	10	20-TGVYRIMTRG-29
	10	52-TTKGAALMSG-61
	10	63-GRLDPYWGSV-72
	10	74-EDRLCYGGPW-83
	12	108-NVQTKPGVFKTP-119
	12	131-PTGTSGSPIVDK-142
	13	145-DVIGLYGNGVIMP-157
	11	161-YISAIVQGERM-171
	12	191-VLDLHPGAGKTR-202
	9	235-ALRGLPIRY-243
	27	256-EIVDVMCHATLTHRLMSPHRVPNYNLF-282
	14	288-HFTDPASIAARGYI-301
	12	310-AAAIFMTATPPG-321
	10	337-QTEIPDRAWN-346
	10	357-GKTVWFVPSV-366
	16	385-QLNRKSYETEYPKCKN-400
	11	408-TTDISEMGANF-418
	11	422-RVIDSRKSVKP-432
	11	451-TAASAAQRRGR-461
	16	470-GDEYCYGGHTNEDDSN-485
	9	487-AHWTEARIM-495
	11	526-LRGEERKNFLE-536
	9	540-TADLPVWLA-548
	10	563-WCFDGPRTNT-572
NS4a	15	19-KTWEALDTMYVVATA-33
	11	43-ALEELPDALQT-53
	13	101-GTKIAGMLLLSLL-113
	20	115-MIVLIPEPEKQRSQTDNQLA-134
NS4b	9	39-PATAWSLYA-47
	13	68-TSLTSINVQASAL-80
	9	85-RGFPFVDVG-93
	12	138-AQRRTAAGIMKN-149
	10	156-VATDVPELER-165
	22	208-VTLWENGASSVWNATTAIGLCH-229
NS5	10	1-GGAKGRTLGE-10
	9	60-AKLRWLVER-68
	17	79-DLGCGRGGWCYYMATQK-95
	22	107-GPGHEEPQLVQSYGWNIVTMKS-128
	10	141-DTLLCDIGES-150
	10	152-SSAEVEEHRT-161
	9	168-VEDWLHRGP-176
	16	208-RNPLSRNSTHEMYWVS-223
	12	235-MTSQVLLGRMEK-246
	10	259-NLGSGTRAVG-268
	13	299-NHPYRTWNYHGSY-311
	18	318-SASSLVNGVVRLLSKPWD-335
	29	340-VTTMAMTDTTPFGQQRVFKEKVDTKAPEP-368
	10	375-VLNETTNWLW-384
	18	404-KVNSNAALGAMFEEQNQW-421
	10	440-EREAHLRGEC-449
	12	451-TCIYNMMGKREK-462
	29	472-GSRAIWFMWLGARFLEFEALGFLNEDHWL-500
	16	504-NSGGGVEGLGLQKLGY-519
	13	533-YADDTAGWDTRIT-545
	10	548-DLENEAKVLE-557
	15	571-IELTYRHKVVKVMRP-585
	23	596-ISREDQRGSGQVVTYALNTFTNL-618
	12	620-VQLVRMMEGEGV-631
	19	662-RMAVSGDDCVVKPLDDRFA-680
	14	689-MSKVRKDIQEWKPS-702
	18	704-GWYDWQQVPFCSNHFTEL-721
	27	741-GRARISPGAGWNVRDTACLAKSYAQMW-767
	21	769-LLYFHRRDLRLMANAICSAVP-789
	12	792-WVPTGRTTWSIH-803

a Numbers prefixing and affixing sequences represent start and end positions in the protein alignment.

**Table 3:** Number of pan-WNV sequences, their length in amino acids and percentage coverage of total protein length.

WNV protein	Total length (aa) a	Pan-WNV sequences		
		Number	Length (aa)	% of total protein length (aa) b
C	123	0	0	0
prM	167	2	24	14
E	497	7	87	18
NS1	352	8	95	27
NS2a	231	2	25	11
NS2b	131	4	44	34
NS3	619	25	296	48
NS4a	149	4	59	40
NS4b	256	6	75	29
NS5	905	30	464	51
Total	3430	88	1169	34

a Approximate length indicated in number of amino acids, according to the reference protein sequence described in the *Methods*.

b Approximate percentage rounded off to nearest whole number.

### Functional and structural analysis of pan-WNV sequences

Sequences conserved throughout the evolutionary history of rapidly mutating RNA viruses are thought to be critical for structure and/or function [36]. A search in the Prosite and Pfam databases [27], [29] revealed that 50 of the 88 pan-WNV sequences are known to be associated with putative or known biological functions and/or structure (**Table 4**); the biological significance of the remaining 38 sequences is still to be determined. In the E protein, two pan-WNV sequences correspond to the fusion loop and dimerisation domain [37], while two correspond to immunoglobulin-like domain, attributed to putative receptor binding sites [38]. One NS1 sequence correspond to the putative ATP/GTP binding site p-loop motif, likely to be involved in helicase activity [39]. NS3 contained 4 pan-WNV sequences that correspond to the peptidase family S7 (*Flavivirus* serine protease) domain [40], and 4 that correspond to known/putative *Flavivirus* Asp-Glu-Ala-Asp/His (DEAD/H) domain associated with ATP-dependent helicase activity [41]. NS5 contained 17 sequences that correspond to the RNA-dependent RNA polymerase (RdRp)/catalytic domain [42], [43]. Furthermore, 33 of the 50 pan-WNV sequences were predicted to exhibit post-translational modification(s), including N-glycosylation, protein kinase C (PKC), casein kinase II (CKII) and tyrosine kinase (TK) phosphorylation, N-myristoylation and/or amidation.

**Table 4:** Reported biological properties of pan-WNV sequences.

WNV protein	Pan-WNV sequence	Functional domains and motifs a	Putative post-transcriptional modifications a
E	1-FNCLGMSNRDF-11	Dimerisation domain	PKC, CKII
	104-GCGLFGKGSIDTCA-117	Dimerisation domain, Fusion Loop	N-myristoylation
	293-LKGTTYGVC-301	-	N-myristoylation
	338-SVASLNDLTPVGRLVTVNP-356	Immunoglobulin-like domain	CKII
	370-ELEPPFGDSYIV-381	Immunoglobulin-like domain	-
	417-LGDTAWDFGS-426	-	CKII
NS1	58-RSVSRLEHQMW-68	-	CKII
	114-GWKAWGKSI-122	ATP/GTP-binding site motif A (P-loop)	-
	209-TWKLERAVLGEVKSCTWPETHTLWG-233	-	PKC, CKII
	328-GCWYGMEIRP-337	-	N-myristoylation
NS2a	69-NSGGDVVHLALMATF-83	-	CKII
NS2b	12-GLMFAIVGGLAELD-25	-	N-myristoylation
NS3	52-TTKGAALMSG-61	-	PKC
	74-EDRLCYGGPW-83	Peptidase S7	-
	108-NVQTKPGVFKTP-119	Peptidase S7	-
	131-PTGTSGSPIVDK-142	Peptidase S7	-
	145-DVIGLYGNGVIMP-157	Peptidase S7	N-myristoylation
	191-VLDLHPGAGKTR-202	DEAD/H domain	N-myristoylation
	256-EIVDVMCHATLTHRLMSPHRVPNYNLF-282	DEAD/H domain	PKC
	288-HFTDPASIAARGYI-301	DEAD/H domain	-
	310-AAAIFMTATPPG-321	DEAD/H domain	-
	385-QLNRKSYETEYPKCKN-400	-	TK
	422-RVIDSRKSVKP-432	-	PKC
	470-GDEYCYGGHTNEDDSN-485	-	CKII, N-myristoylation
NS4a	101-GTKIAGMLLLSLL-113	-	N-myristoylation
	115-MIVLIPEPEKQRSQTDNQLA-134	-	CKII
NS4b	208-VTLWENGASSVWNATTAIGLCH-229	-	N-glycosylation, CKII
NS5	1-GGAKGRTLGE-10	-	CKII, N-myristoylation
	79-DLGCGRGGWCYYMATQK-95	-	PKC, N-myristoylation
	107-GPGHEEPQLVQSYGWNIVTMKS-128	-	PKC
	152-SSAEVEEHRT-161	-	CKII
	208-RNPLSRNSTHEMYWVS-223	-	N-glycosylation, CKII
	299-NHPYRTWNYHGSY-311	RdRp	-
	318-SASSLVNGVVRLLSKPWD-335	RdRp	-
	340-VTTMAMTDTTPFGQQRVFKEKVDTKAPEP-368	RdRp	-
	375-VLNETTNWLW-384	-	N-glycosylation
	404-KVNSNAALGAMFEEQNQW-421	RdRp	-
	451-TCIYNMMGKREK-462	RdRp	Amidation
	472-GSRAIWFMWLGARFLEFEALGFLNEDHWL-500	RdRp	-
	504-NSGGGVEGLGLQKLGY-519	RdRp	N-myristoylation
	533-YADDTAGWDTRIT-545	RdRp	-
	571-IELTYRHKVVKVMRP-585	RdRp	PKC
	596-ISREDQRGSGQVVTYALNTFTNL-618	RdRp/ RdRp catalytic domain	CKII, N-myristoylation
	620-VQLVRMMEGEGV-631	RdRp	-
	662-RMAVSGDDCVVKPLDDRFA-680	RdRp/ RdRp catalytic domain	CKII
	689-MSKVRKDIQEWKPS-702	RdRp	-
	704-GWYDWQQVPFCSNHFTEL-721	RdRp	-
	741-GRARISPGAGWNVRDTACLAKSYAQMW-767	RdRp	N-myristoylation
	769-LLYFHRRDLRLMANAICSAVP-789	RdRp	-
	792-WVPTGRTTWSIH-803	RdRp	PKC

a Prosite (PS) and Pfam (PF) accession numbers: PS00001, N-glycosylation site; PS00005, Protein kinase C phosphorylation (PKC) site; PS00006, Casein kinase II (CKII) phosphorylation site; PS00007, Tyrosine kinase (TK) phosphorylation site; PS00008, N-myristoylation site; PS00009, Amidation site; PS00017, ATP/GTP-binding site motif A (P-loop); PS50507, RNA-directed RNA polymerase (RdRp) catalytic domain; PF00869, dimerisation domain; PF00949, Peptidase S7; PF00972, RNA-directed RNA polymerase (RdRp); PF02832, Immunoglobulin-like domain; PF07652, *Flavivirus* DEAD/H domain.

Amino acid residues exposed and protruding on the surface of viral proteins are generally subject to fewer packing constraints and residue interactions as compared to those buried within protein interiors. Thirty of the 88 pan-WNV sequences could be mapped on available, but incomplete, WNV protein structures obtained from the PDB (E protein, 2HG0; NS3, 2IJO; and NS5, 2HFZ) (**Figure S1**). Five pan-WNV sequences were mostly buried and an equal number of pan-WNV sequences were partially exposed (13) or largely exposed (12). These results should be considered preliminary until full-length 3-D structures are available.

### Distribution of pan-WNV sequences in nature

**Figure 4:**
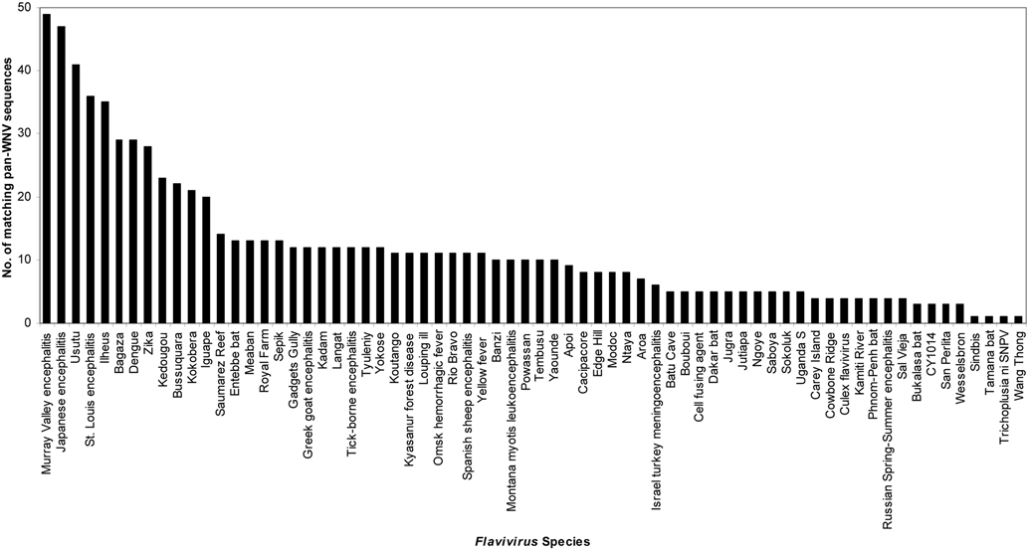
Number of pan-WNV sequences conserved in other flaviviruses.

**Figure 5:**
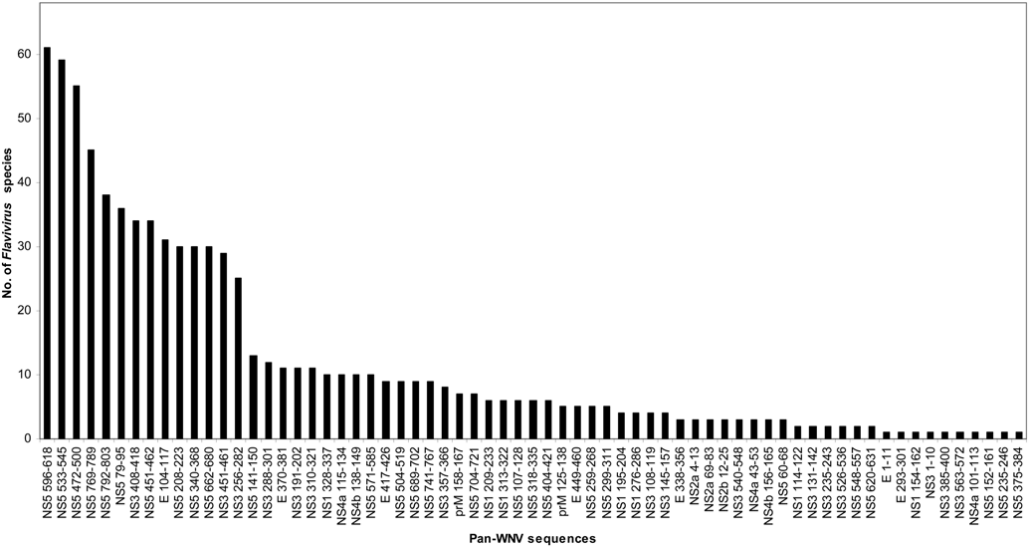
Number of flaviviruses shared by the pan-WNV sequences.

Sixty-seven (67) of the 88 pan-WNV sequences (∼76%) overlapped at least 9 amino acid sequences of as many as 68 other viruses of the family *Flaviviridae*, genus *Flavivirus* (**Figure 4**). Each of these 67 sequences matched at least one, and at most 61 *Flavivirus* species (**Figure 5** **and Table S1**). Murray valley encephalitis (MVE) virus shared 49 of the 67 pan-WNV sequences; JEV and Usutu viruses shared 47 and 41, respectively; and representatives of some of the important human pathogens, St. Louis encephalitis (LEV), DENV, YFV, and tick-borne encephalitis (TBEV) viruses shared from 36 to 11 of the 67 pan-WNV sequences. The representation (frequency) of these pan-WNV sequences ranged from low to high across reported sequences of the several well studied flaviviruses, including DENV, JEV, Louping ill (LIV), Omsk hemorrhagic fever (OMSK), Powassan (PV), LEV, TBEV, and YFV (**Table S1**). For example, the pan-WNV sequence E_104–117_ was present in 99% of the 245 E protein JEV sequences, while E_293–301_ was present in only 1% of the 256 E protein JEV sequences.

Fifty-eight (58) of the 67 pan-WNV sequences shared by other flaviviruses were from the non-structural proteins. Of the 27 pan-WNV sequences found in NS5, 10 were present in at least 30 *Flavivirus* species; while of the 16 sequences in NS3, three were found in between 25 and 34 other species; The remaining 15 sequences were contained in non-structural proteins NS1 (7), NS2a (2), NS2b (1), NS4a (3) and NS4b (2). Nine (9) of the 67 pan-WNV sequences shared by flaviviruses originated from the structural proteins E (7) and prM (2); one of the E protein sequences was present in 31 species.

Remarkably, 5 of the 88 pan-WNV sequences (prM_158–167_, NS3_408–418_, NS4b_208–229_, NS5_1–10_, and NS5_504–519_) shared 9 consecutive amino acids with 7 non-viral species. The nonamer sequence from prM_158–166_ is found in the bacterium *Acidiphilium cryptum* JF-5; NS3_409–417_ in the mosquito *Aedes albopictus*; NS4b_218–226_ in the Japanese rice *Oryza sativa* (japonica cultivar-group); NS5_2–10_ in the bacterium *Actinomyces odontolyticus*, NS5_504–512_ in the bacteria *Burkholderia ambifaria* MC40-6 and *Burkholderia cepacia* AMMD; and NS_506–514_ in the bacterium *Methylobacterium extorquens* PA1.

### Known and predicted HLA supertype-restricted, pan-WNV T-cell epitopes

**Figure 6:**
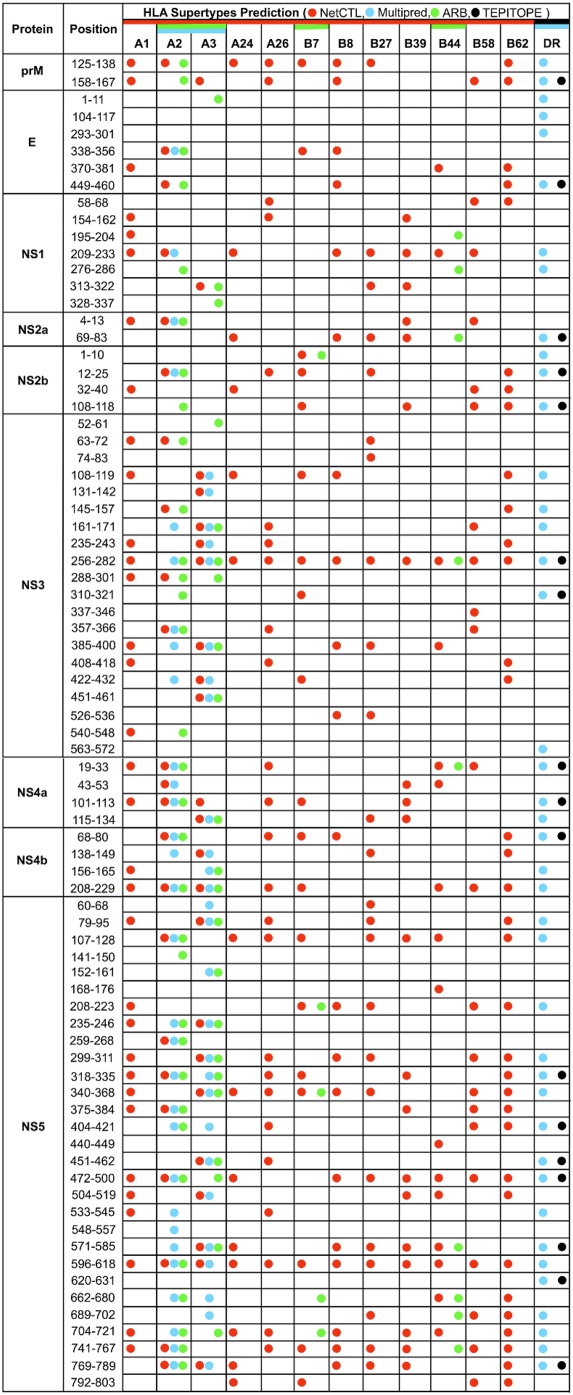
Putative HLA supertype-restricted, pan-WNV T-cell epitopes predicted by computational algorithms.

A literature survey and IEDB database search revealed that 3 of the pan-WNV sequences (2 in NS3, and one in NS5) matched 3 previously reported WNV T-cell epitopes immunogenic in human, having HLA restriction (when known), with both class I (B*07) and II (DR2) specificities (**Table 5**). Further evaluation of the immune-relevance of pan-WNV sequences included a search for putative promiscuous HLA supertype-restricted T-cell epitopes within these regions by use of NetCTL, Multipred, ARB and TEPITOPE prediction tools. Seventy-eight (78) of the 88 pan-WNV sequences (∼89%) were predicted to contain 271 supertype-restricted binding nonamers (**Figure 6** and **Table S2**). Of these sequences, 62 contained nonamers predicted to bind to multiple HLA supertypes. Clusters of predicted binders, two or more overlapping nonamer peptides with identical HLA supertype-restrictions, known as hotspots [33], [44], were found in 41 of the 78 sequences. Seven (7) of the 78 sequences had at least 3 sequential nonamers overlapping by 8 amino acids. As these sequences are completely conserved, all of these epitopes are found in all reported WNV strains.

**Table 5:** WNV sequences with human T-cell epitopes elucidated by other studies.

WNV protein	Pan-WNV sequence	Reported T-cell epitopes immunogenic in humans			
		Sequence a	T-cells	HLA restriction	Reference(s) b
NS3	145-DVIGLYGNGVIMP-157	VIGLYGNGV	CD4	DR2	[50]
	256-EIVDVMCHATLTHRLMSPHRVPNYNLF-282	SPHRVPNYNL	CD8	B07	[51]
NS5	704-GWYDWQQVPFCSNHFTEL-721	FCSNHFTEL	-	-	1021472

a Epitope amino acids matching the pan-WNV sequences are underlined.

b 1021472 is an accession number of a record in the Immune Epitope Database.

In addition, 44 pan-WNV sequences were found to contain sequences of at least 9 amino acids present in 54 CD4^+^CD8^−^ and/or CD4^−^CD8^+^ IFN-γ ELISpot positive peptides (**Table 6**), identified by peptide-specific T-cell responses of murine H-2 class I or II-deficient transgenic mice, expressing prototypic class I HLA-A2 (A*0201), -A24 (A*2402) and -B7 (B*0702), and class II HLA-DR2 (DRB1*1501), -DR3 (DRB1*0301) and -DR4 (DRB1*0401) alleles, and immunized with overlapping peptides covering the entire WNV proteome (unpublished data of Jung KO *et al.*, of Johns Hopkins University, Maryland, USA). Twenty-three (23) of the 44 pan-WNV sequences that overlapped the ELISpot positive peptides correspond to positive HLA-DR supertype-restricted T-cell epitope predictions by either Multipred or TEPITOPE (**Table 6** **and Table S2**). The experimental data revealed that 11 out of 44 pan-WNV sequences, localized in prM, E, NS1, NS3, NS4a, NS4b and NS5, were promiscuous for at least two HLA-DR alleles; the promiscuity of 9 of these 11 pan-WNV sequences were correctly predicted (**Table S2**). In summary, combined with previously reported data for human WNV T-cell epitopes from literature and public database (**Table 5**), at least 44 of the 88 pan-WNV sequences contained numerous HLA-restricted class I and/or class II epitopes demonstrated by *in vivo* T-cell response assays.

**Table 6:** Pan-WNV sequences with human T-cell epitopes identified by use of HLA transgenic mice.

WNV protein	Pan-WNV sequence a	T-cell epitopes immunogenic in HLA transgenic mice	
		ELISpot activation peptide b	ELISpot positive HLA transgenic mouse
prM	125-ESWILRNPGYALVA-138^*^	LVKTESWILRNPGYALVA	DR2 & DR4
		LRNPGYALVAAVIGWML	A24, B7 & DR2
	158-LLLLVAPAYS-167^*^	RVVFVVLLLLVAPAYS	A2, DR2, DR3 & DR4
E	104-GCGLFGKGSIDTCA-117^*^	RGWGNGCGLFGKGSI	DR3 & DR4
	293-LKGTTYGVC-301^*^	EKLQLKGTTYGVCSKAFK	DR4
	370-ELEPPFGDSYIV-381	KVLIELEPPFGDSYIVV	DR4
	449-LFGGMSWITQGL-460^*^	FRSLFGGMSWITQGLLGA	A2, DR2 & DR3
NS1	209-TWKLERAVLGEVKSCTWPETHTLWG-233^*^	RLNDTWKLERAVLGEVK	DR4
	276-DFDYCPGTTVT-286^*^	EGRVEIDFDYCPGTTVTL	DR4
	313-CRSCTLPPLR-322	GKLITDWCCRSCTLPPLR	DR3 & DR4
	328-GCWYGMEIRP-337	SGCWYGMEIRPQRHDEK	DR4
NS2a	69-NSGGDVVHLALMATF-83^*^	FAESNSGGDVVHLALMA	DR4
NS2b	1-GWPATEVMTA-10^*^	GWPATEVMTAVGLMFAIV	DR4
	108-SAYTPWAILPS-118^*^	ISAYTPWAILPSVVGFWI	A24, B7 & DR4
NS3	1-GGVLWDTPSP-10	GGVLWDTPSPKEYKK	B7 & DR4
	52-TTKGAALMSG-61	WHTTKGAALMSGEGRL	DR3
	074-EDRLCYGGPW-083	GSVKEDRLCYGGPWKLQH	A2
	145-DVIGLYGNGVIMP-157	PIVDKNGDVIGLYGNGVI	A2
		VIGLYGNGVIMPNGSYI	A2
	161-YISAIVQGERM-171^*^	YISAIVQGERMDEPIPA	A2 & DR2
	191-VLDLHPGAGKTR-202	MLRKKQITVLDLHPGAGK	A2 & DR2
		VLDLHPGAGKTRRILPQI	DR2
	235-ALRGLPIRY-243	VAAEMAEALRGLPIRY	DR4
		EALRGLPIRYQTSAVPR	DR4
	256-EIVDVMCHATLTHRLMSPHRVPNYNLF-282^*^	PREHNGNEIVDVMCHATL	A2, DR2 & DR4
		IVDVMCHATLTHRLMSPH	DR2
		TLTHRLMSPHRVPNYNLF	A2 & DR2
	310-AAAIFMTATPPG-321	KVELGEAAAIFMTATPPG	A2
	337-QTEIPDRAWN-346	LQTEIPDRAWNSGYEWI	A2
	422-RVIDSRKSVKP-432	EMGANFKASRVIDSRKSV	A2
	470-GDEYCYGGHTNEDDSN-485	CYGGHTNEDDSNFAHW	A2 & DR3
	487-AHWTEARIM-495	AHWTEARIMLDNINM	A2 & DR3
	526-LRGEERKNFLE-536	EYRLRGEERKNFLELLR	A2 & DR2
	563-WCFDGPRTNT-572^*^	DRRWCFDGPRTNTIL	DR3
NS4a	19-KTWEALDTMYVVATA-33^*^	HFMGKTWEALDTMYVVA	DR2 & DR4
	115-MIVLIPEPEKQRSQTDNQLA-134^*^	VLIPEPEKQRSQTDNQLA	DR4
NS4b	39-PATAWSLYA-47	GEFLLDLRPATAWSLYAV	DR2
		PATAWSLYAVTTAVLTPL	DR2 & DR3
	68-TSLTSINVQASAL-80^*^	DYINTSLTSINVQASALF	DR3 & DR4
	208-VTLWENGASSVWNATTAIGLCH-229^*^	LITAAAVTLWENGASSVW	DR3 & DR4
NS5	107-GPGHEEPQLVQSYGWNIVTMKS-128^*^	LVQSYGWNIVTMKSGVDV	DR3
	152-SSAEVEEHRT-161	CDIGESSSSAEVEEHRTI	B7
		SAEVEEHRTIRVLEMV	A2, B7 & DR2
	208-RNPLSRNSTHEMYWVS-223^*^	SRNSTHEMYWVSRASGNV	DR2
	451-TCIYNMMGKREK-462	ECHTCIYNMMGKREKK	A2
	472-GSRAIWFMWLGARFLEFEALGFLNEDHWL-500	AKGSRAIWFMWLGARFL	A24
		WFMWLGARFLEFEALGFL	A24
	596-ISREDQRGSGQVVTYALNTFTNL-618^*^	REDQRGSGQVVTYALNTF	DR2
		GQVVTYALNTFTNLAVQL	DR2 & DR4
	620-VQLVRMMEGEGV-631^*^	NTFTNLAVQLVRMMEGEGV	DR4
	704-GWYDWQQVPFCSNHFTEL-721^*^	GWYDWQQVPFCSNHFTEL	DR4
	741-GRARISPGAGWNVRDTACLAKSYAQMW-767	DTACLAKSYAQMWLLLYF	A24
	769-LLYFHRRDLRLMANAICSAVP-789^*^	YAQMWLLLYFHRRDLRLM	B7 & DR4
	792-WVPTGRTTWSIH-803	NWVPTGRTTWSIHAGGEW	DR4

a Pan-WNV sequences that are predicted, either by Multipred or TEPITOPE, to contain at least one HLA-DR supertype-restricted binding nonamer are indicated by an asterisk (*).

b Epitope amino acids matching the pan-WNV sequences are underlined.

## Discussion

In the 70 years following the discovery of WNV in Africa in 1937 [45], there has been 100% conservation of 88 pan-WNV sequences, corresponding collectively to 1169 aa or ∼34% of the 3,430 aa total composition of the viral proteome. The remaining 66% of the proteome contained one or more amino acid variants within each nonamer segment across the reported WNV sequences. Most of the pan-WNV sequences were found in the non-structural proteins. Quantitatively, 40% (1058/2643 aa) of the amino acids of the non-structural proteins (NS1, NS2a, NS2b, NS3, NS4a, NS4b and NS5) comprised the pan-WNV sequences, compared to only 14% (111/787 aa) of the structural proteins (C, prM and E). This marked difference in the evolutionary conservation/variability of the viral proteins can be attributed to greater demands on the integrity of nonstructural proteins in their viral functional roles, and possibly to the selective advantage of modified structural proteins in the adaptation to host immune responses. This evolutionary history of the conserved protein sequences extends to other members of the *Flaviviridae* family, with 67 of the 88 pan-WNV sequences shared among at least 68 other flaviviruses. Many of the identified critical biological and/or structural properties are associated with the conserved sequences; for example, the E dimerisation domain and fusion loop [37], [38], NS3 peptidase S7, DEAD/H domain [40], [41], and NS5 proteins RdRp domain [42], [43]. Hence, these conserved sequences are unlikely to significantly diverge in newly emerging WNV isolates in the future, and represent attractive targets for the development of diagnostics, specific anti-viral compounds and vaccine candidate targets. In short, they can be defined as multi-purpose immutable, functional and immunological tags of WNV.

It is also noteworthy that 9 consecutive amino acids of 5 of the pan-WNV sequences are also present in non-viral proteomes, the *Aedes albopictus* mosquito, *Oryza sativa* Japanese rice and several bacteria. This overlap of pan-WNV sequences with non-viral sequences is possibly coincidental, but is likely to be statistically significant as the probability of randomly matching a nonamer is almost negligible (1/(20^9^)). WNV protein sequences found in the proteomes of bacteria are possibly due to integration of some unknown virus into the bacterial genome [46], [47].. Similarly, the NS3 nonamer sequence fragment found in the Asian Tiger mosquito (*Aedes albopictus*), is possibly due to genetic recombination between phyla [48]. Unexpectedly, a nonamer of WNV NS4b protein was found in a single instance within a plant pathogenesis-related protein from Japanese rice (*Oryza sativa*), which functions as plant defense system against pathogens [49].

There is evidence that many of the conserved sequences are immunologically relevant in humans. Numerous (44/88) contained at least 9 amino acids overlapping with a total of 54 peptides that have been reported to be immunogenic in humans and/or HLA Tg mice. In addition, putative T-cell epitopes were predicted by computational analysis for 12 major HLA class I supertypes and for class II DR supertype, with broad application to the immune responses of human population worldwide. Some of the putative T-cell epitopes were predicted to be promiscuous to multiple HLA supertypes as has been observed with several viruses [17]. These findings of the limited variability of WNV sequences relevant to cellular immunity point to the probable success in the development of a WNV vaccine as compared to the history of failure of candidate vaccines against the much more highly variable *Flavivirus*, such as DENV [17].

A comparison can be made with a similar study of each of the four DENV serotypes [17]. In contrast to WNV, the sequences of the combined serotypes of DENV are highly diverse, with only 44 pan-DENV sequences, representing 15% of the proteome length, that are present in 80% or more of the sequences of each serotype; only two of these 44 sequences were completely conserved in all the four serotypes. However, the conservation and variability of each DENV serotype is comparable to WNV. The individual DENV serotypes and the WNV show remarkable stability over the entire recorded history of their sequences, as demonstrated by their low peptide entropies and variant frequencies. The pan-WNV sequences matched sequences of DENV (one or more serotypes) with representations ranging from low (3%) to high (100%); similar observations were made for pan-DENV sequences matching WNV sequences (4 to 100%). The conserved sequences that matched with low representation may pose potential risk of altered ligands resulting in pathologic immune responses following co-infection or vaccination and secondary infection with a similar virus. Thus, while the consequences of such extensive possible cross-reactive immunity are hypothetical, we propose, for vaccine formulation, that it is prudent to select conserved sequences that are specific to the pathogen, and thus representative of a minimal number of variant sequences.

## Supporting Information

Figure S1 The localization of pan-WNV sequences (in purple) on the three dimensional structure of the respective WNV proteins (E - 2HG0, NS3 - 2IJO and NS5 - 2HFZ). Abbreviations: (E) major portion exposed, (P) partially exposed, (B) major portion buried.
(12.01 MB DOC)

Table S1 Percentage representation of pan-WNV sequences in other flaviviruses.
(0.12 MB DOC)

Table S2 Putative HLA supertype-restricted binding nonamer peptides in pan-WNV sequences, predicted by immunoinformatics algorithms (NetCTL, Multipred (MP), ARB and TEPITOPE (TP)).
(0.79 MB DOC)

Data Set S1 GI numbers of West Nile virus proteins downloaded from the NCBI Entrez Protein Database.
(0.12 MB XLS)
