# Supplementary material for: Conservation and Variability of West Nile Virus Proteins
Source: PLoS One. 2009 Apr 29;4(4):e5352. doi: 10.1371/journal.pone.0005352 (PMC2670515; doi:10.1371/journal.pone.0005352)
Supplement: Figure S1 — The localization of pan-WNV sequences (in purple) on the three dimensional structure of the respective WNV proteins (E - 2HG0, NS3 - 2IJO and NS5 - 2HFZ). Abbreviations: (E) major portion exposed, (P) partially exposed, (B) major portion buried. (12.01 MB DOC) [file pone.0005352.s001.doc]

**Figure S1**

**1a**

**1b**

**2a**

**2b**

**3a**

**3b**

**4a**

**4b**

**
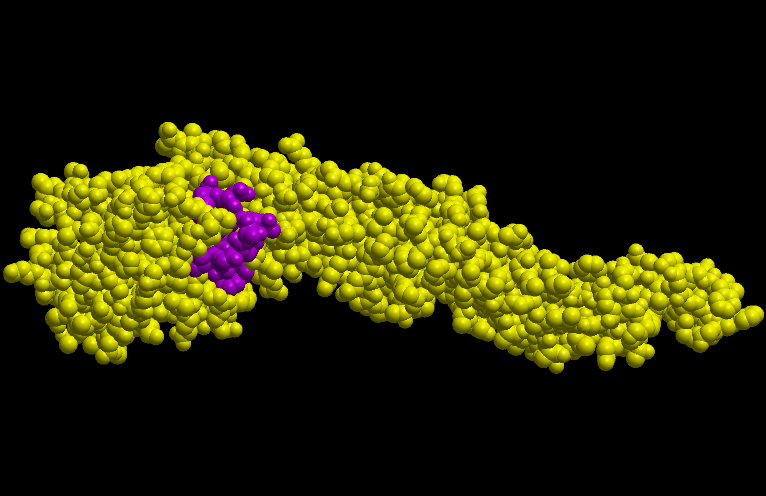

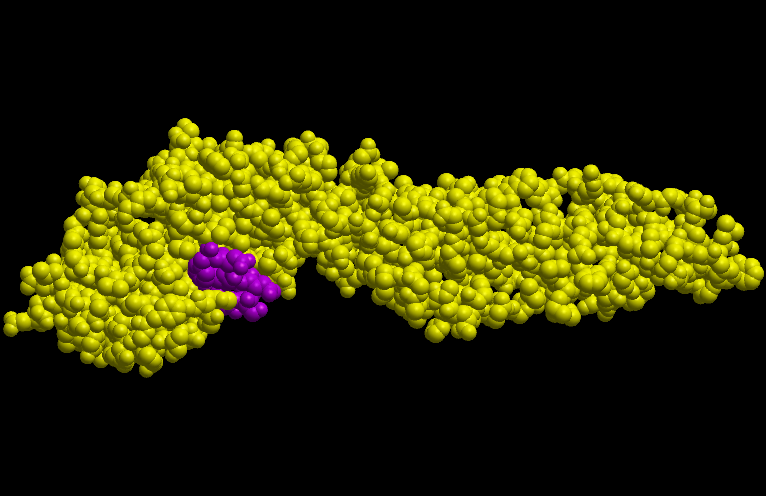

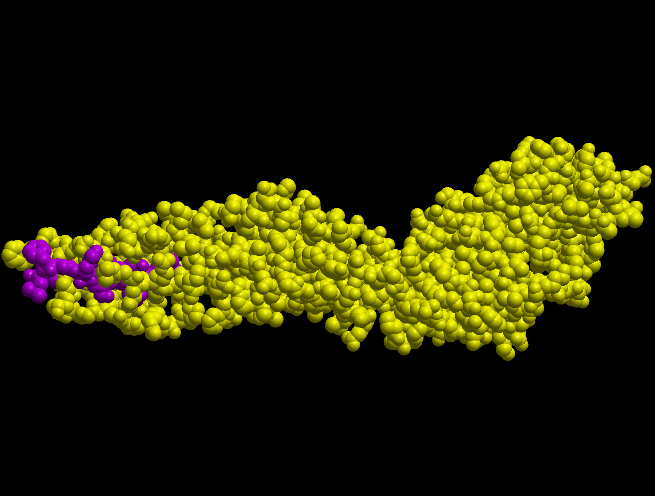

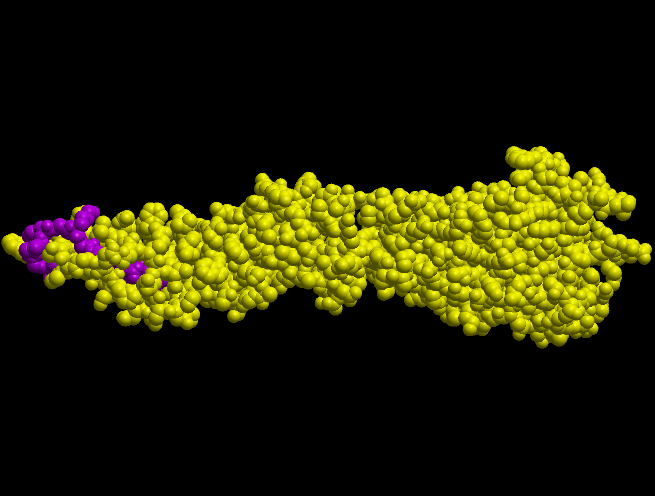

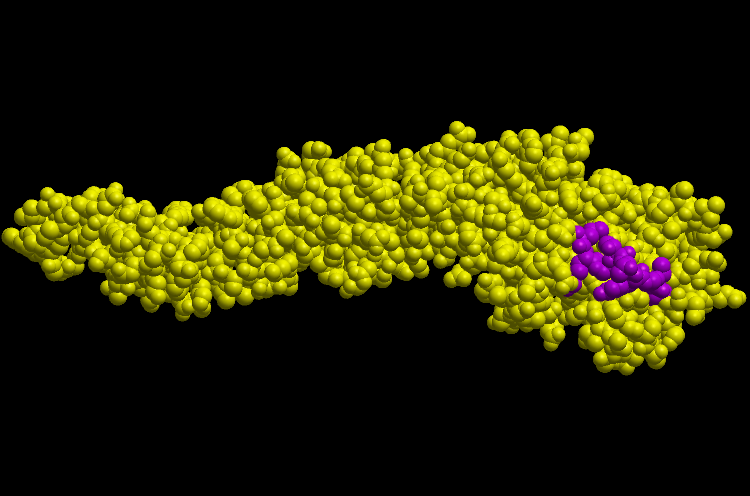

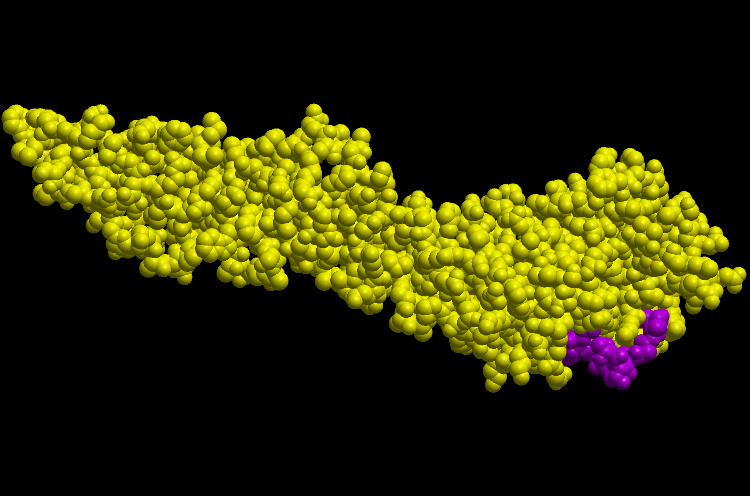

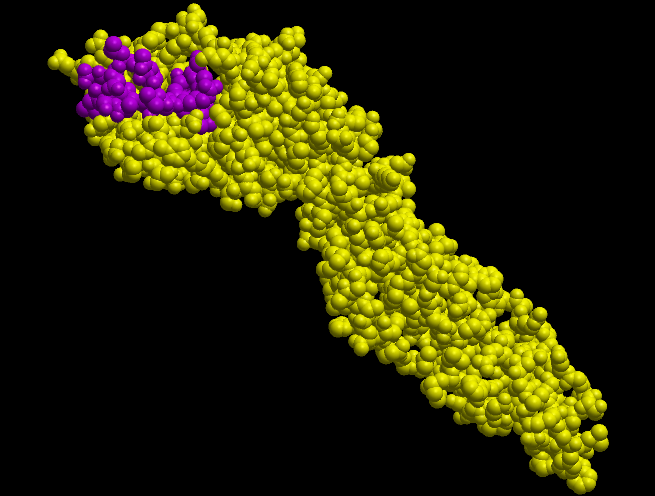

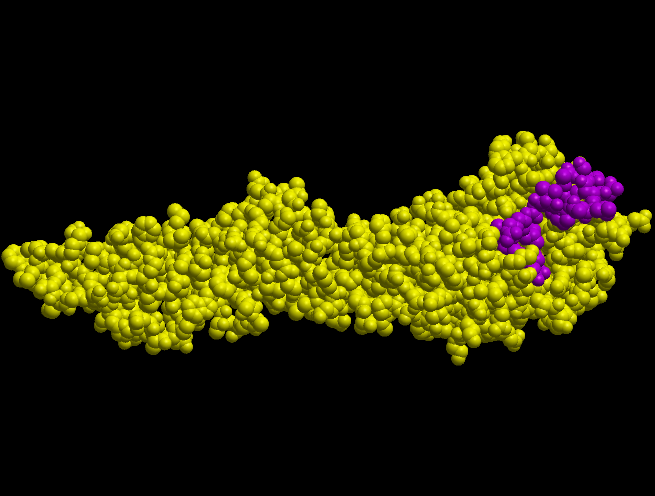
**

1. E_1-11_ (E) | 2. E_104-117_ (P) | 3. E_293-301_ (E) | 4. E_338-356_ (E)

**5a**

**5b**

**6a**

**6b**

**7a**

**7b**

**8a**

**8b**

**9a**

**9b**

**
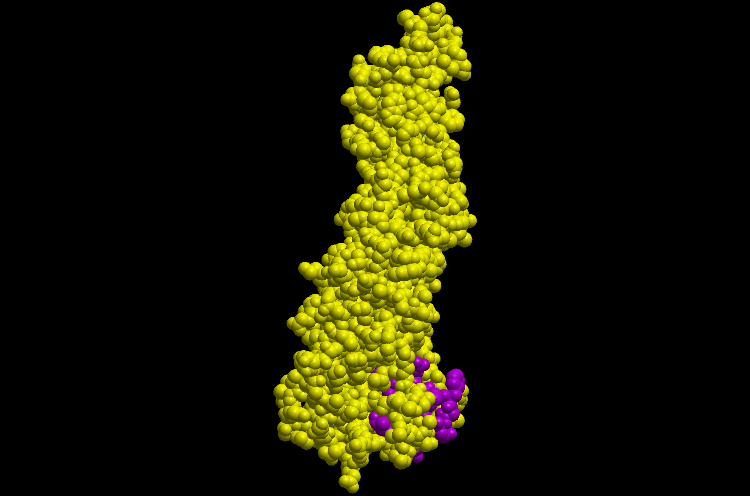

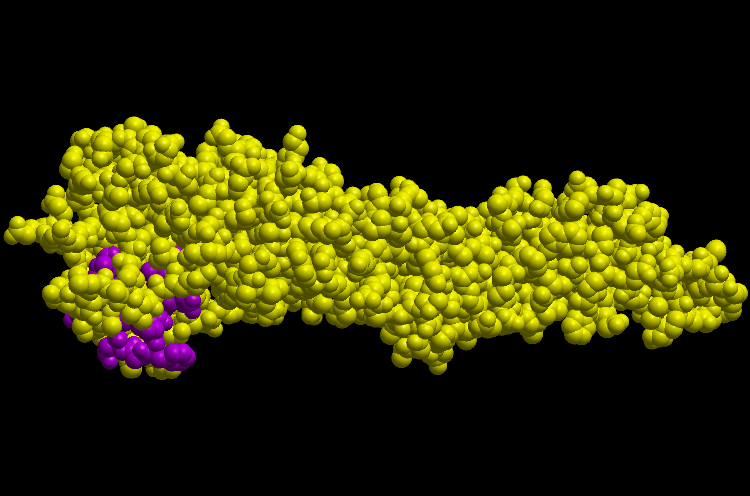

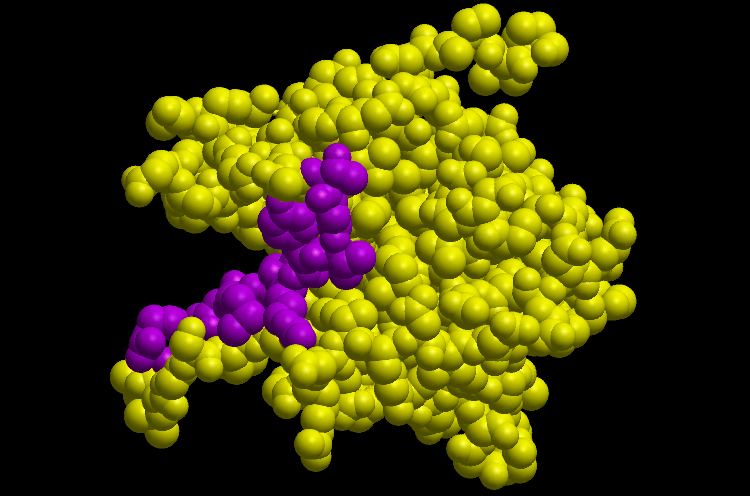

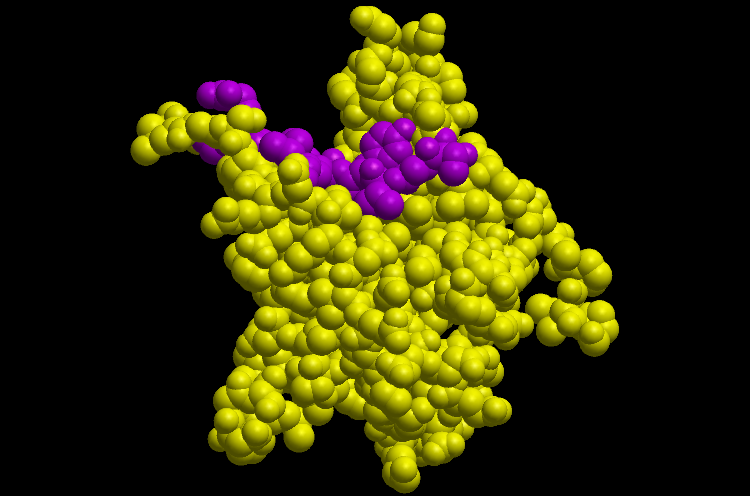

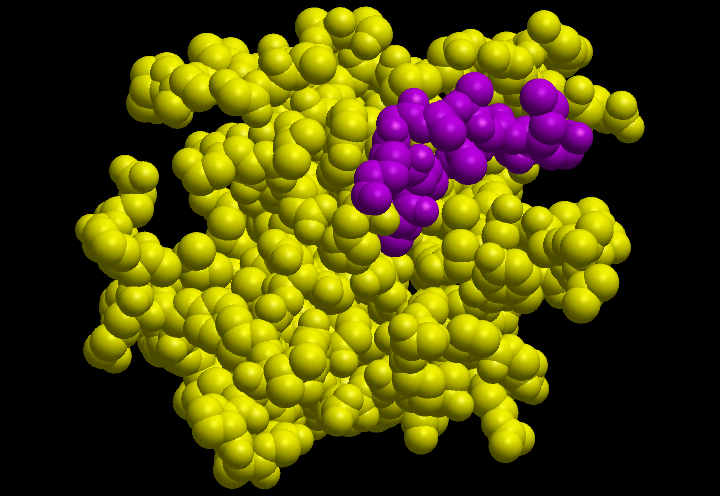

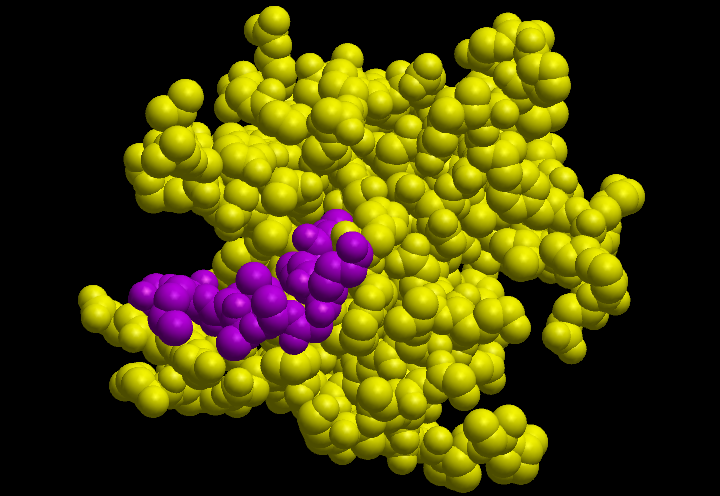

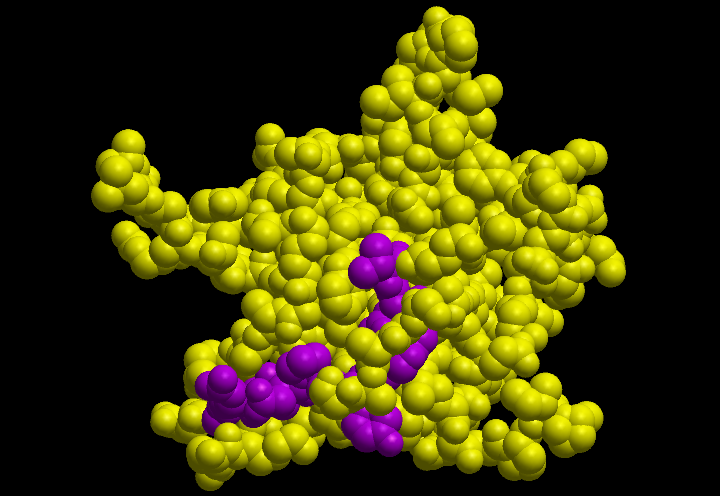

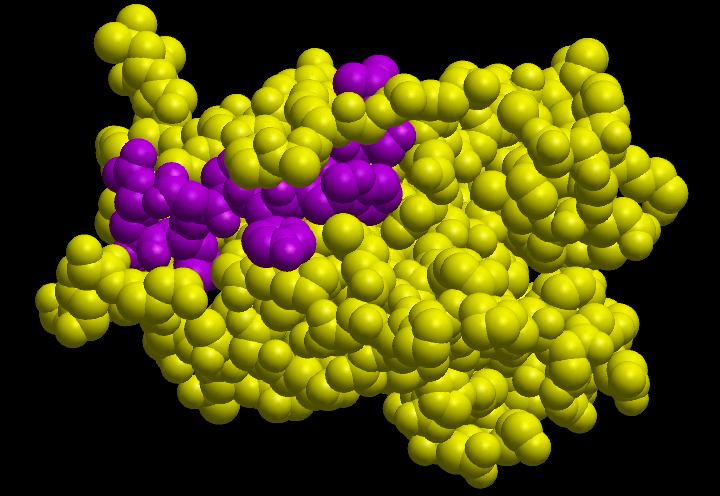

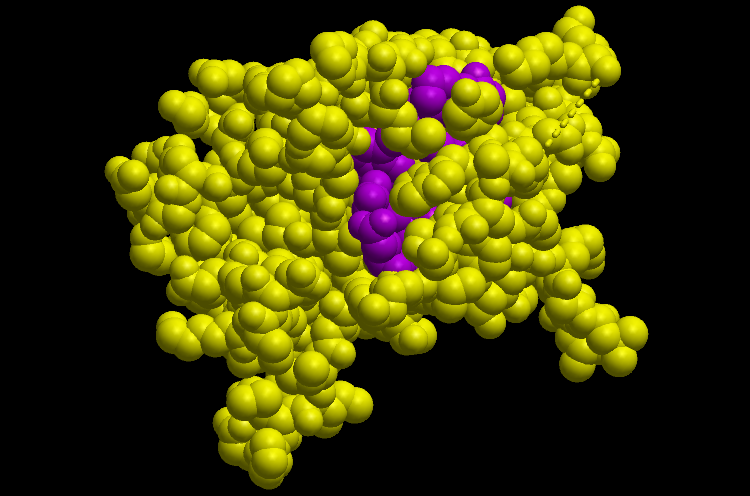

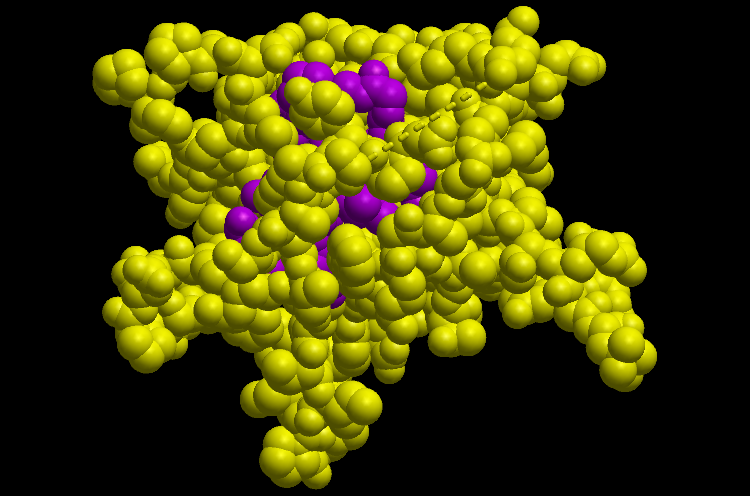
**

5. E_370-381_ (P) | 6. NS3_20-29_ (E) | 7. NS3_52-61_ (E) | 8. NS3_63-72_ (P) | 9. NS3_74-83_ (B)

10. NS3_108-119_ (E) | 11. NS3_131-142_ (B) | 12. NS3_145-157_ (P) | 13. NS3_161-171_ (P) | 14. NS5_318-335_ (E)

**10a**

**10b**

**11a**

**11b**

**12a**

**12b**

**13a**

**13b**

**14a**

**14b**

**
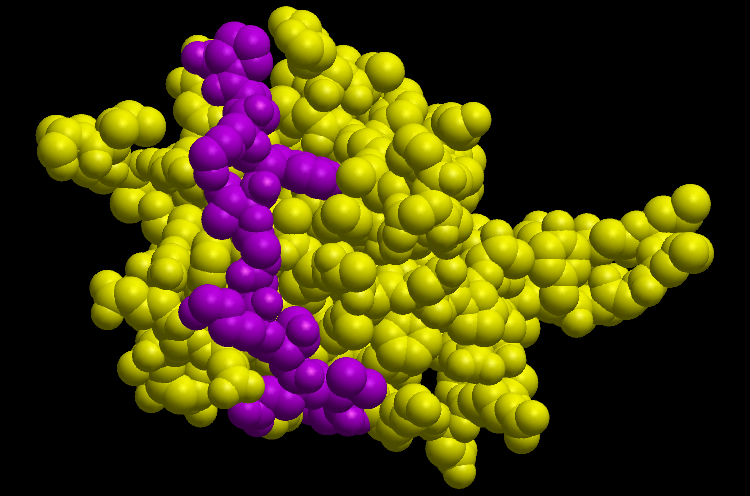

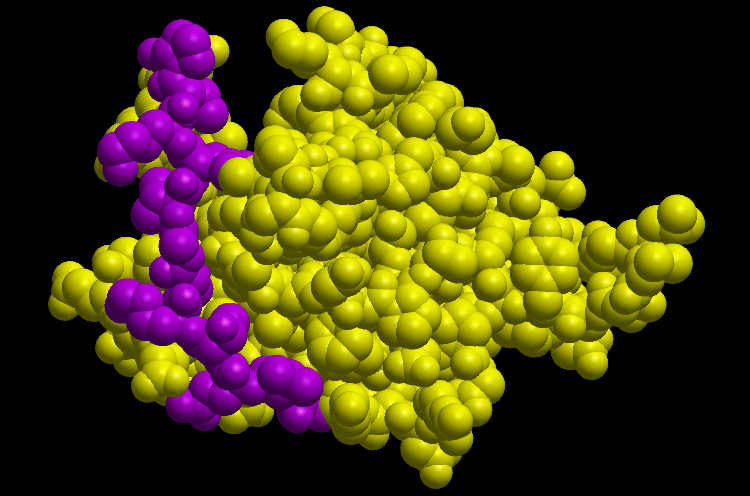

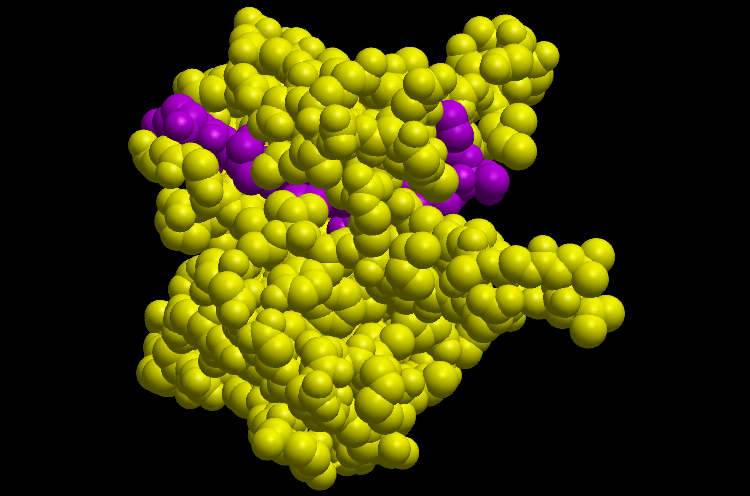

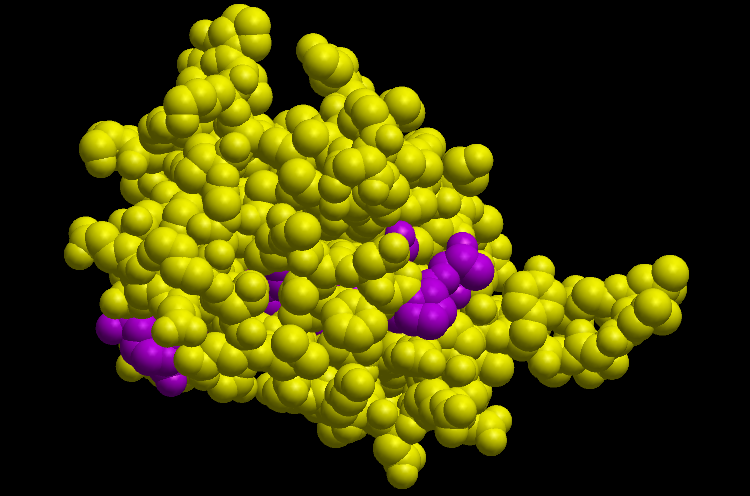

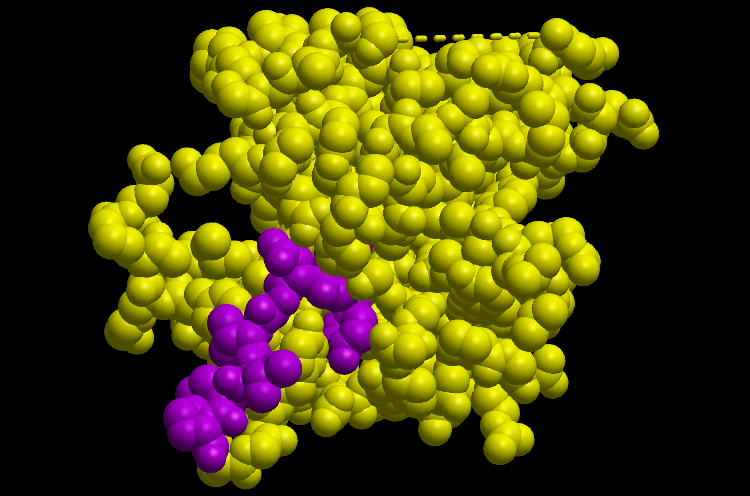

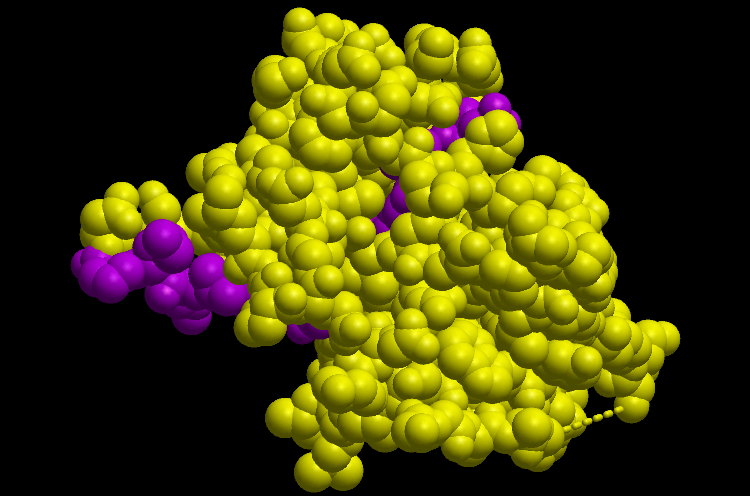

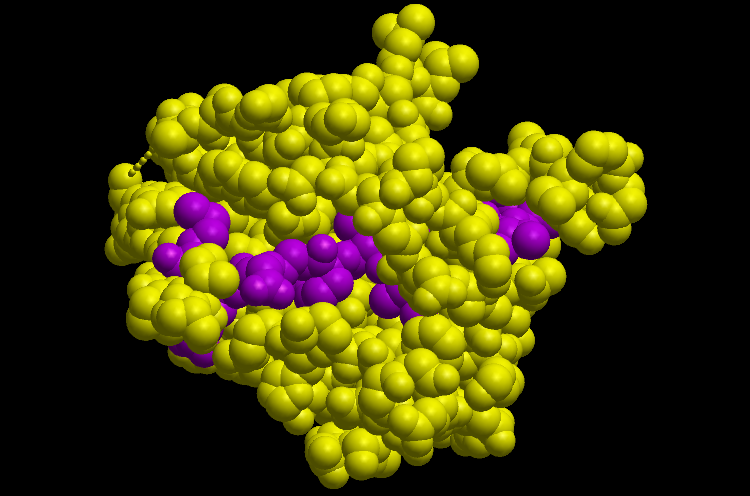

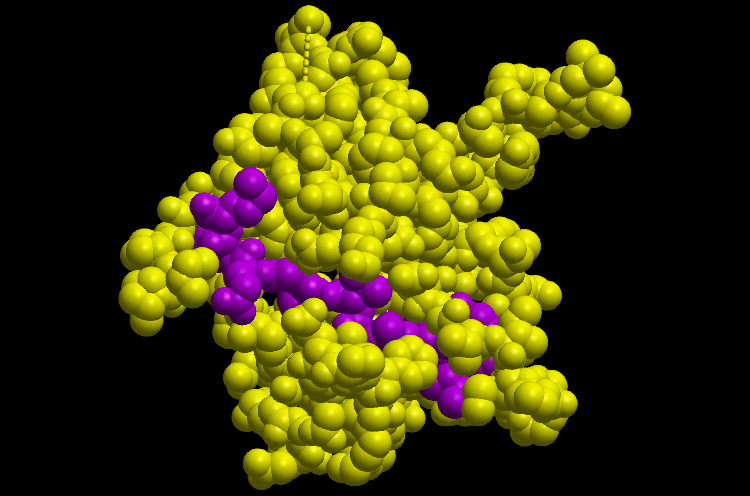

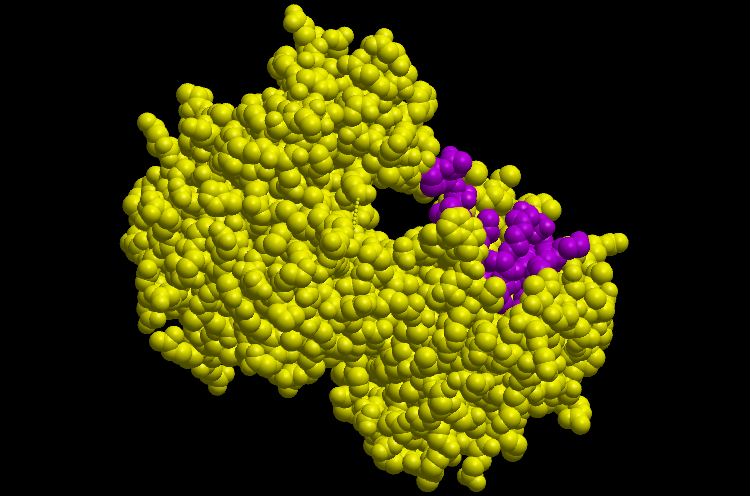

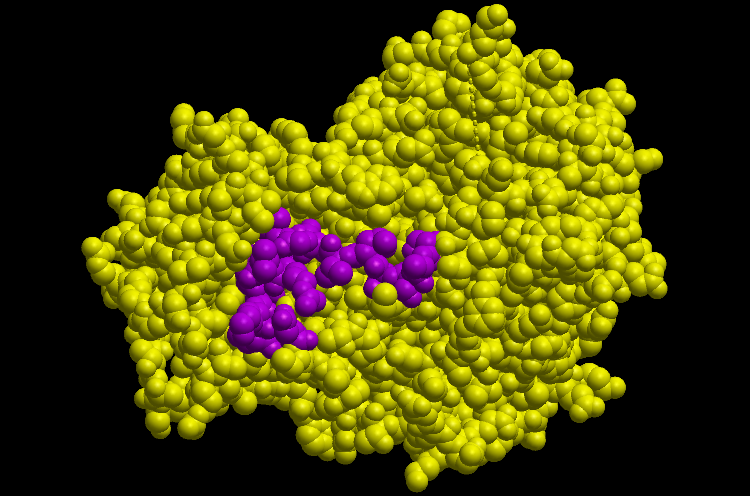
**

15. NS5_340-368_ (E) | 16. NS5_375-384_ (P) | 17. NS5_440-449_ (E) | 18. NS5_472-500_ (P) | 19. NS5_504-519_ (P)

**15a**

**15b**

**16a**

**16b**

**17a**

**17b**

**18a**

**18b**

**19a**

**19b**

**
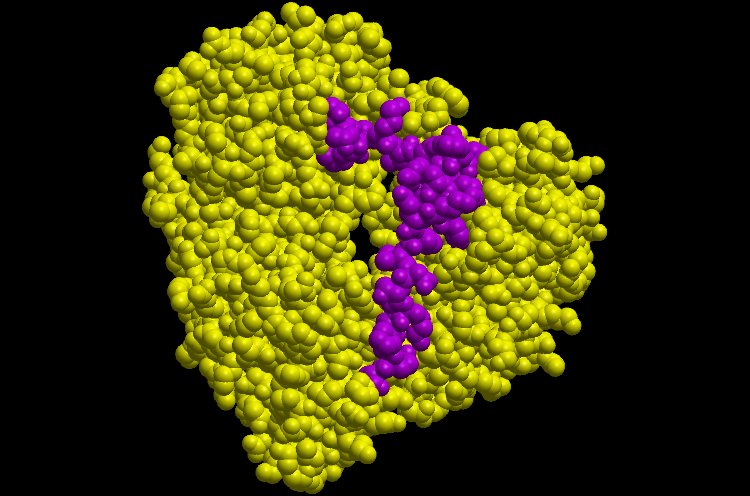

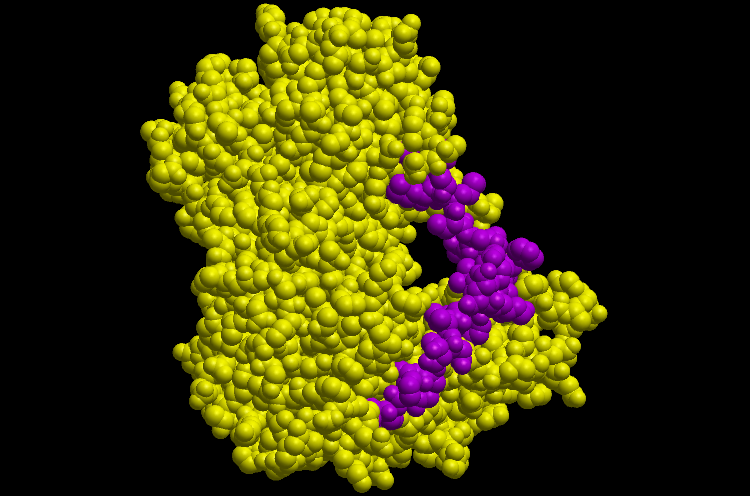

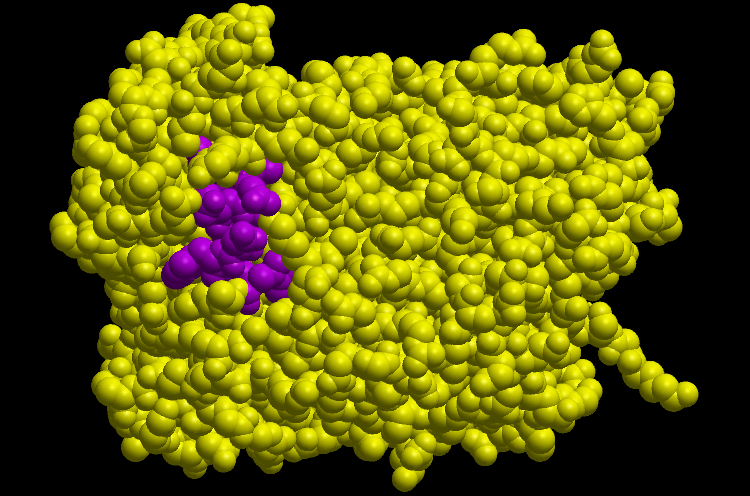

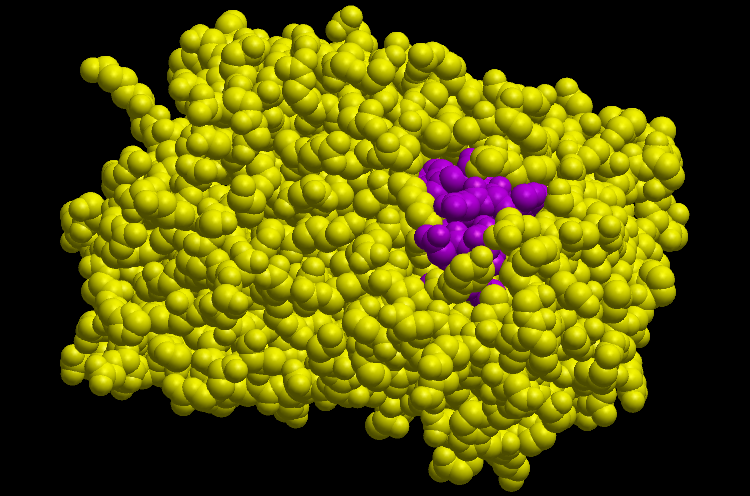

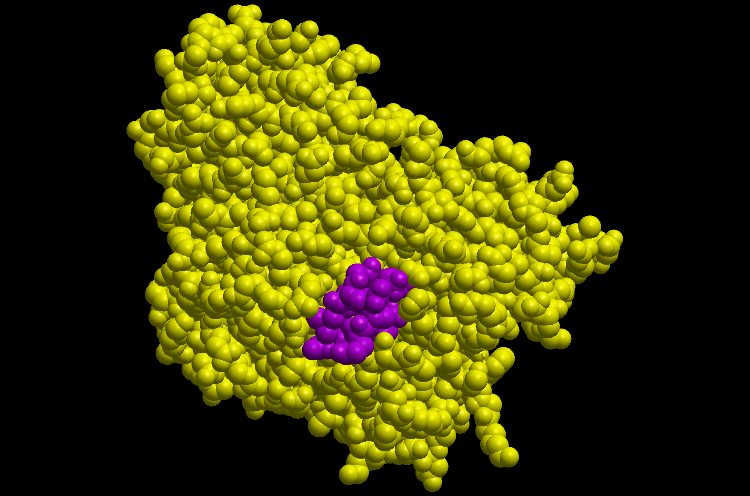

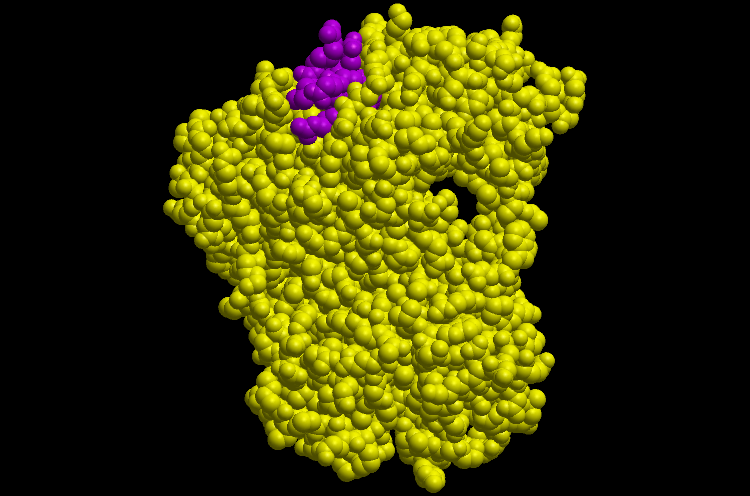

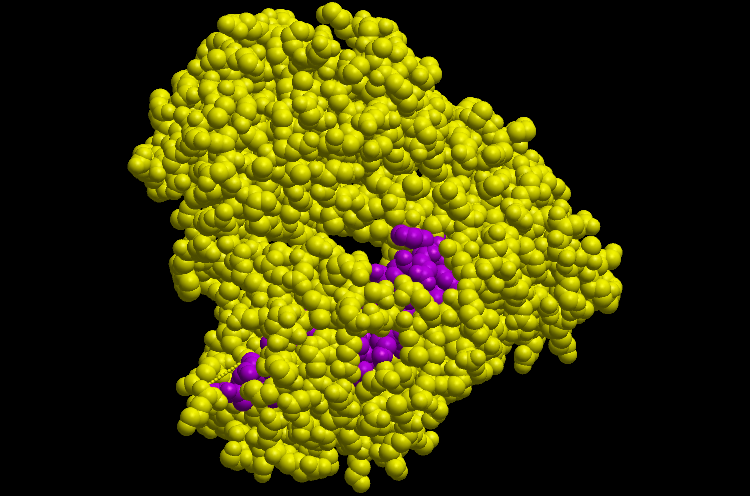

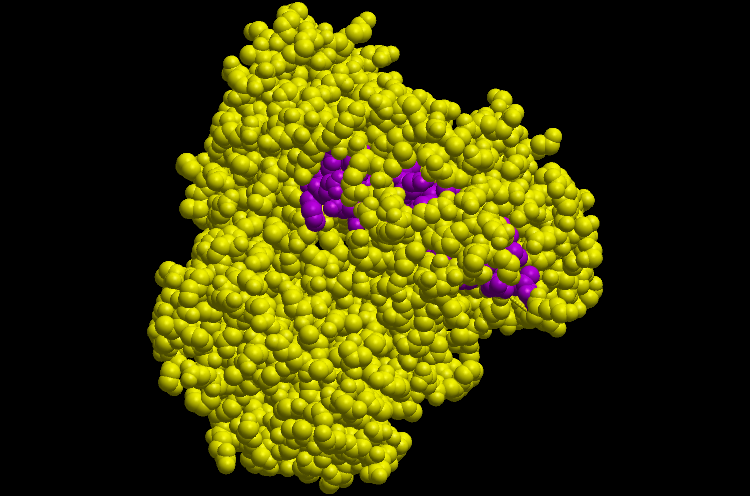

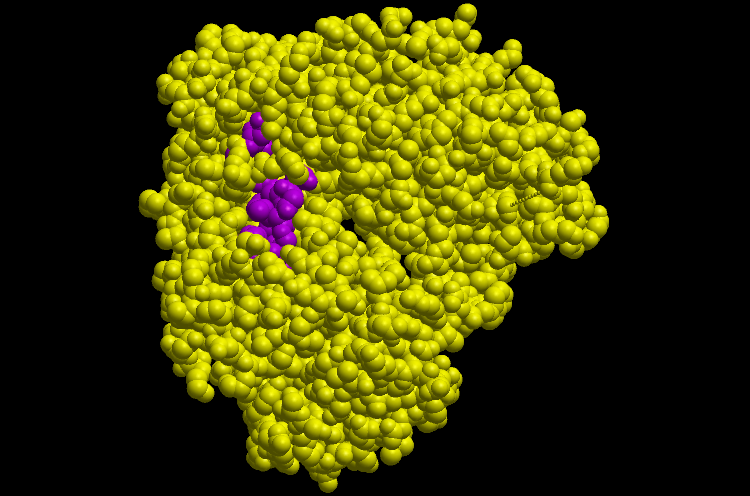

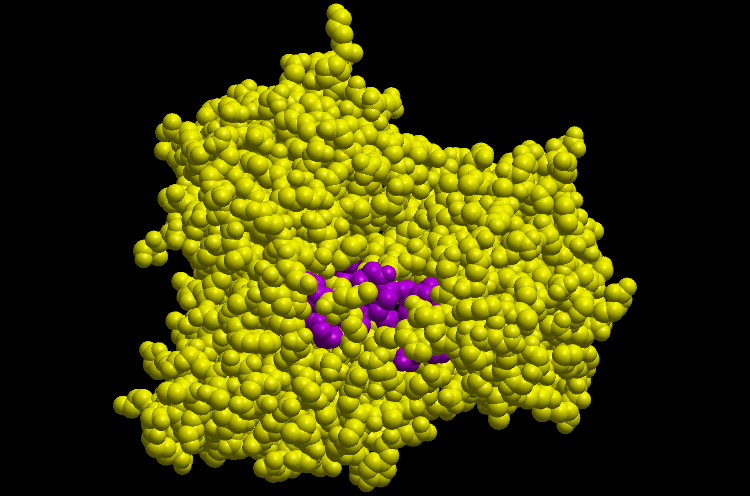
**

20. NS5_533-545_ (P) | 21. NS5_548-557_ (E) | 22. NS5_571-585_ (B) | 23. NS5_596-618_ (P) | 24. NS5_620-631_ (B)

**20a**

**20b**

**21a**

**21b**

**22a**

**22b**

**23a**

**23b**

**24a**

**24b**

**
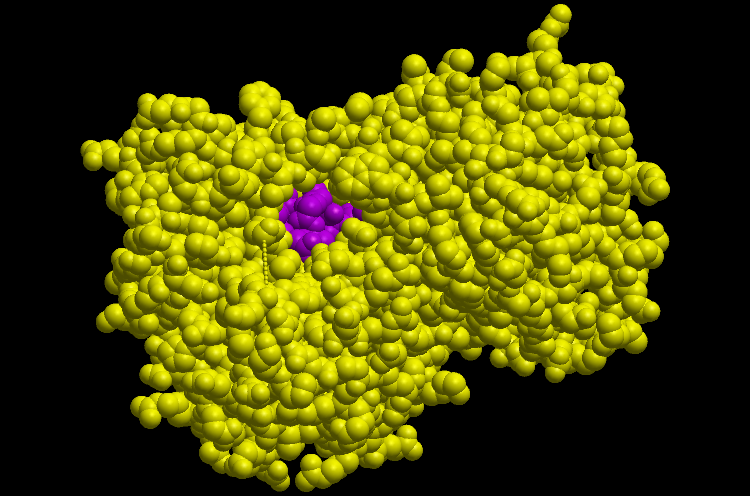

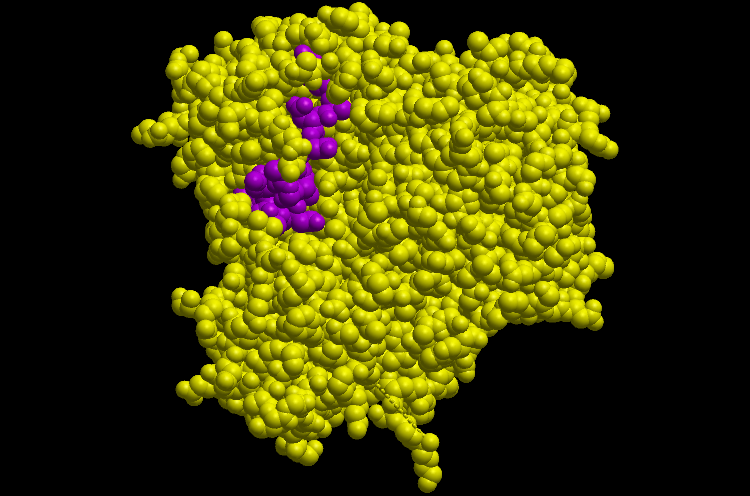

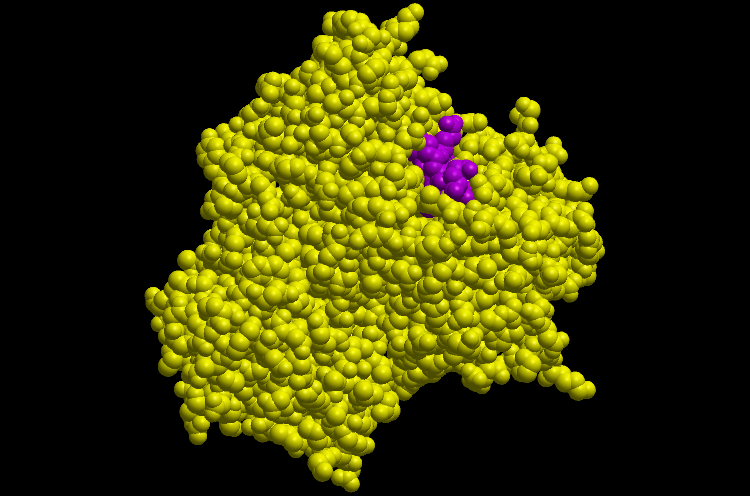

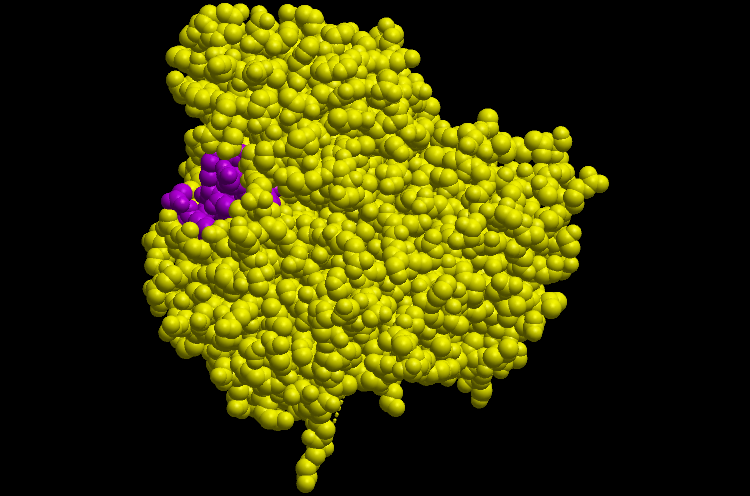

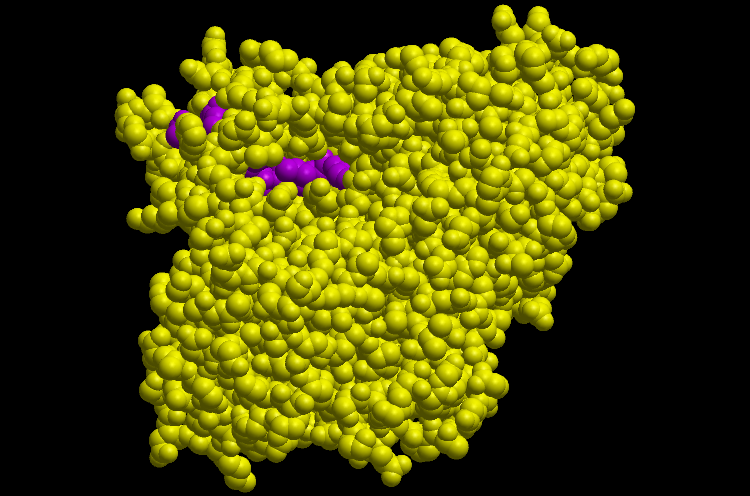

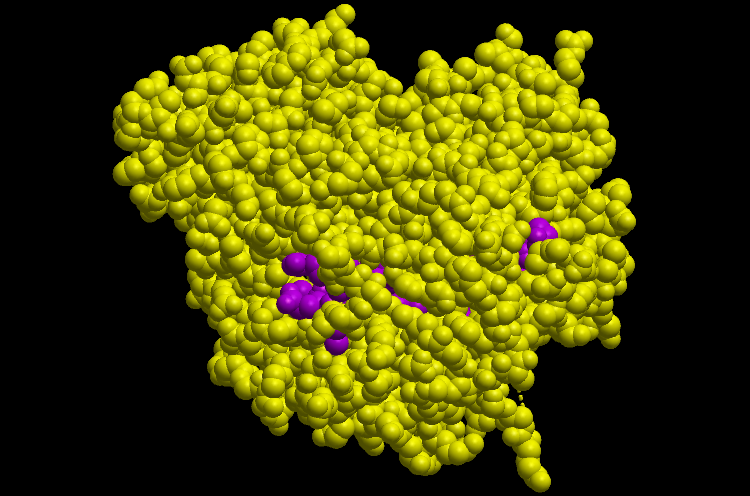

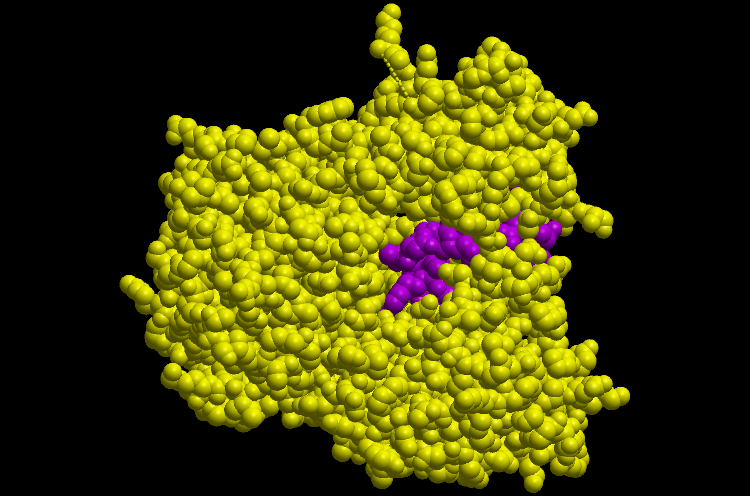

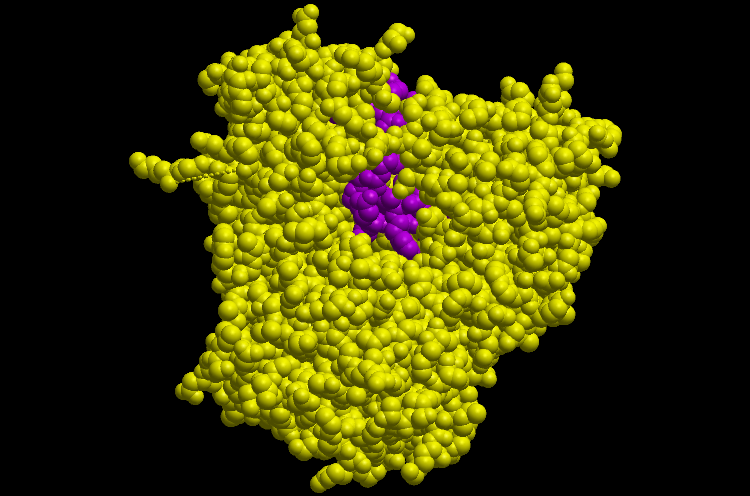

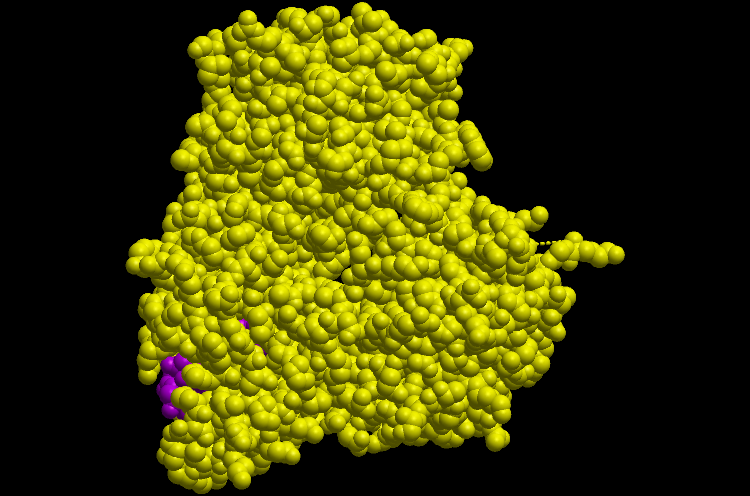

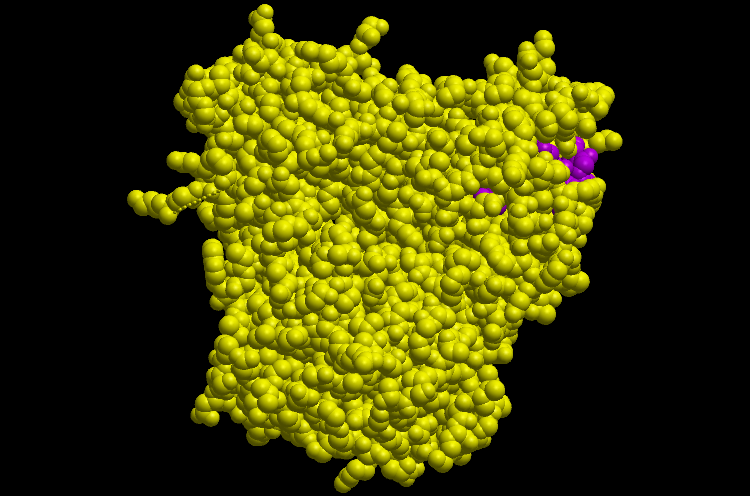
**

25. NS5_662-680_ (P) | 26. NS5_689-702_ (E) | 27. NS5_704-721_ (E) | 28. NS5_741-767_ (P) | 29. NS5_769-789_ (B)

**25a**

**25b**

**26a**

**26b**

**27a**

**27b**

**28a**

**28b**

**29a**

**29b**

**
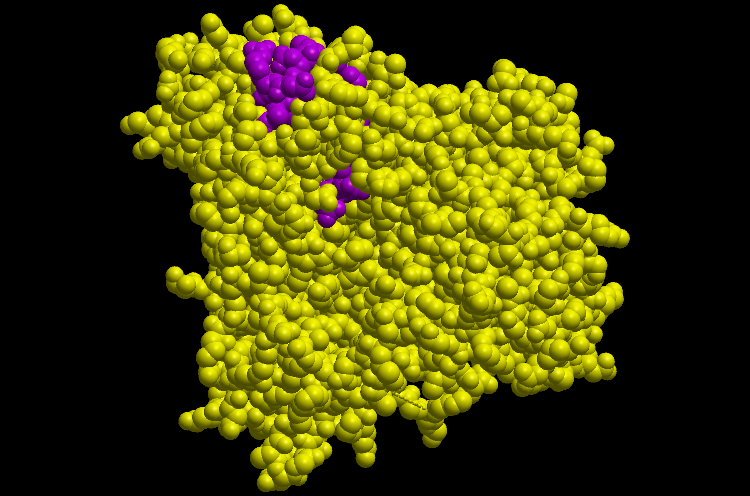

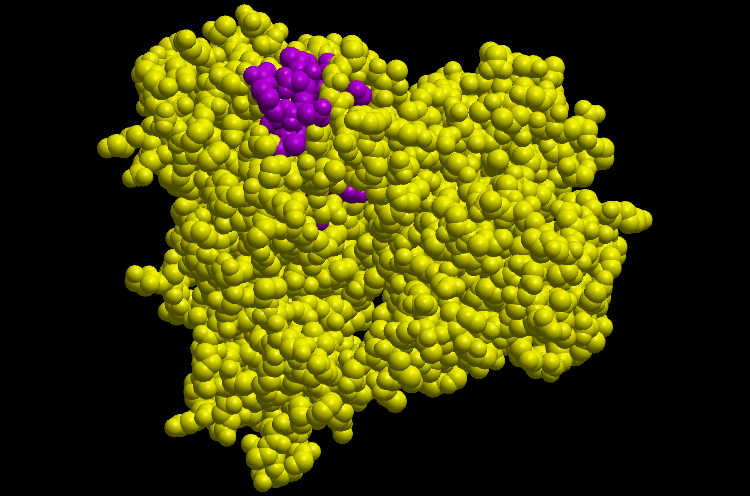

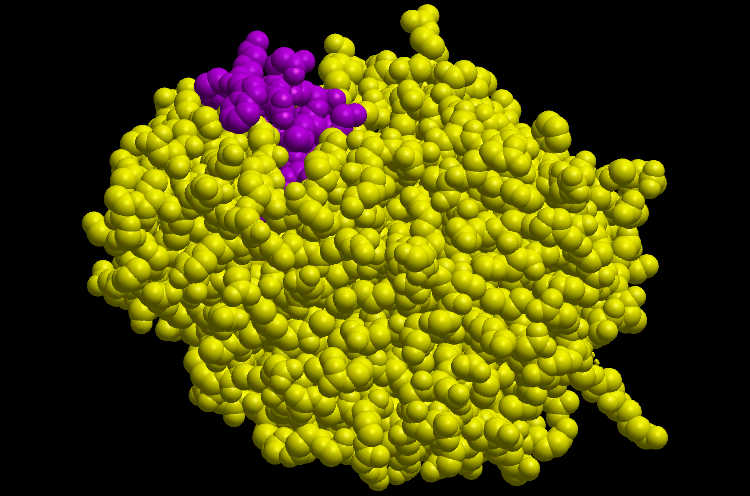

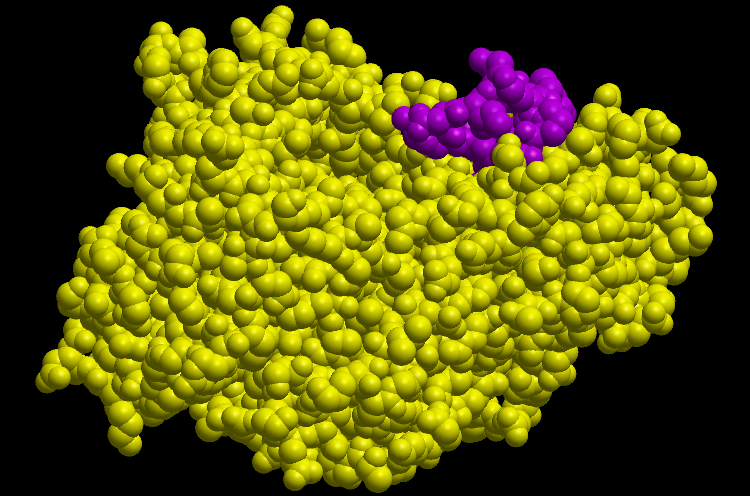

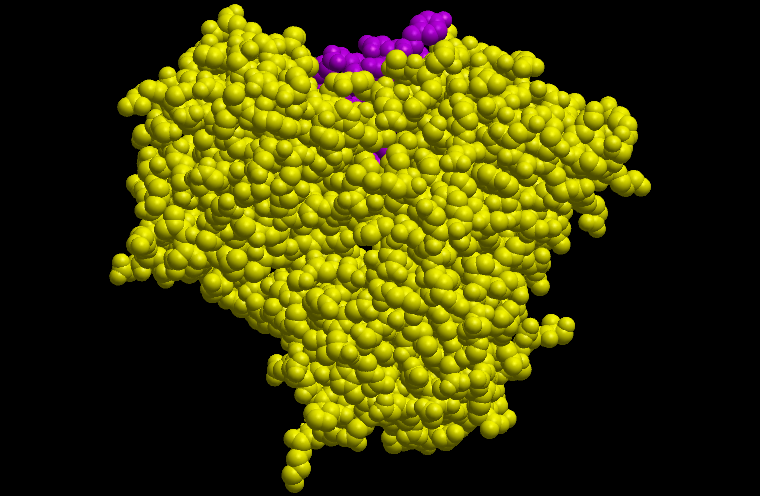

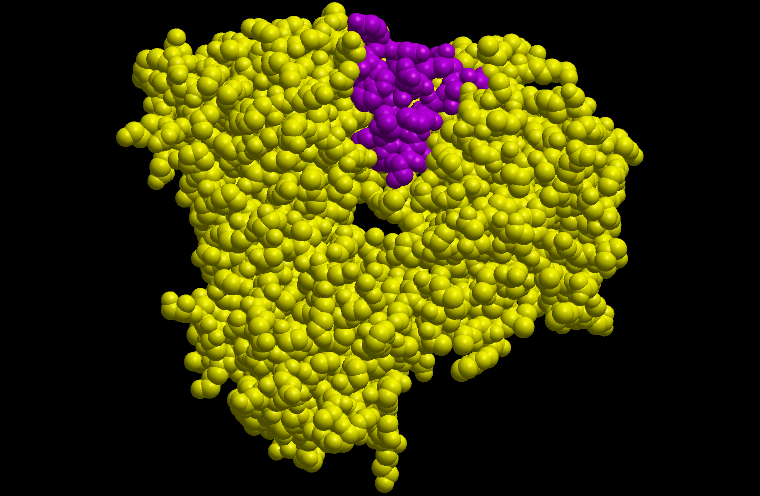

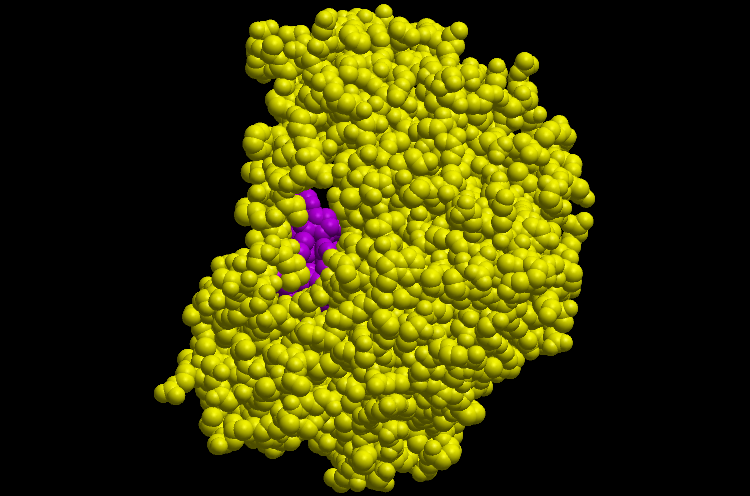

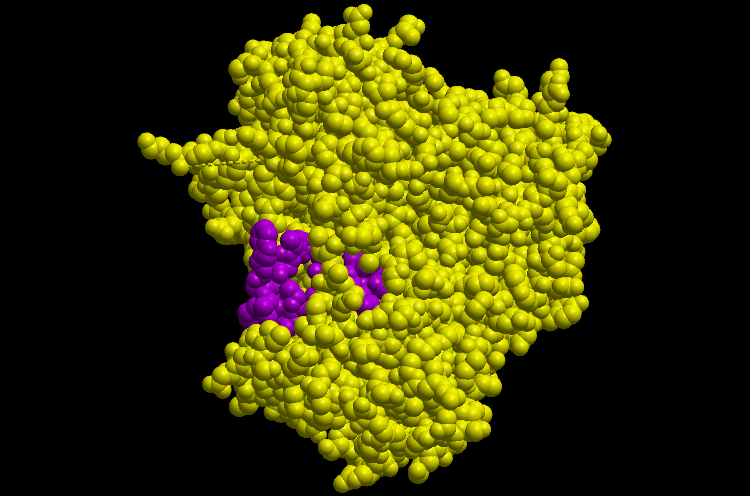

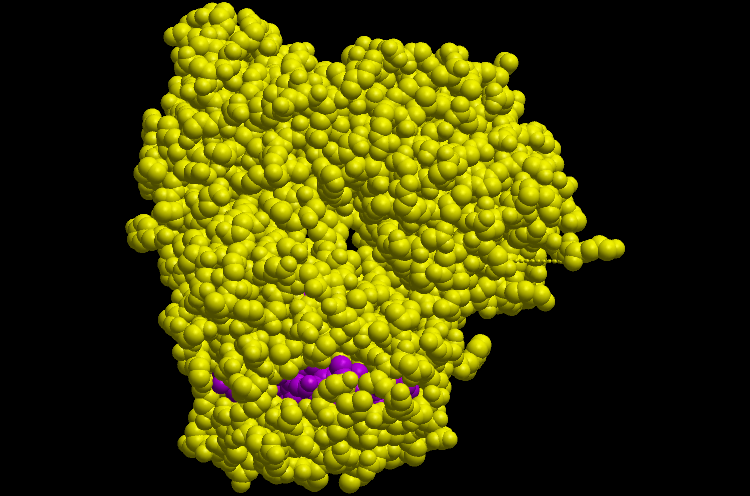

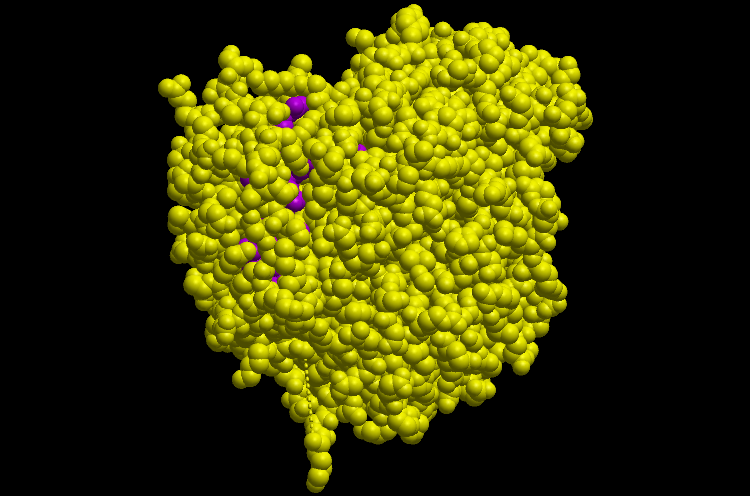
**

**30a**

**30b**

**
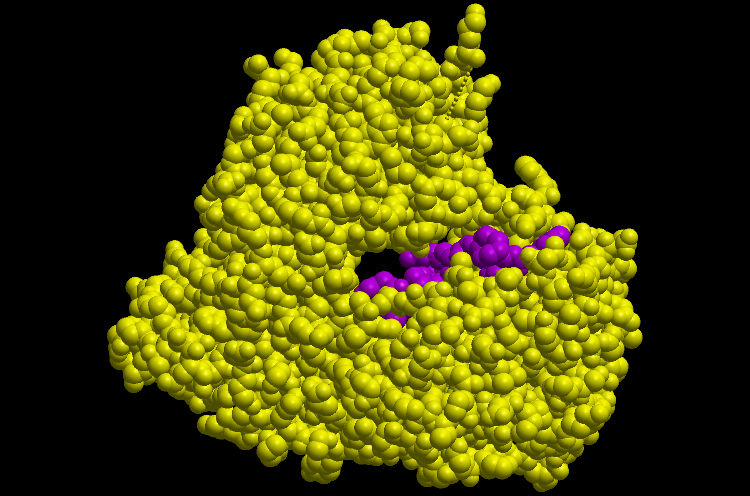

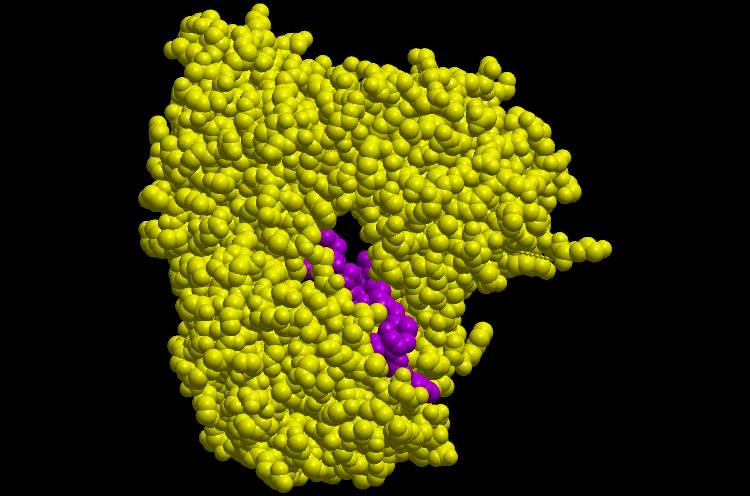
**

30. NS5_792-803_ (P)
