# Supplementary material for: Conservation and Variability of West Nile Virus Proteins
Source: PLoS One. 2009 Apr 29;4(4):e5352. doi: 10.1371/journal.pone.0005352 (PMC2670515; doi:10.1371/journal.pone.0005352)
Supplement: Table S1 — Percentage representation of pan-WNV sequences in other flaviviruses. (0.12 MB DOC) [file pone.0005352.s002.doc]

**Table S1.**

| WNV  protein | Pan-WNV sequence | Species  (#) *^a^* | Percentage representation (%) | Total number of sequences analyzed *^b^* | | | | | | | |
| --- | --- | --- | --- | --- | --- | --- | --- | --- | --- | --- |
|  |  |  | DENV | JEV | LIV | OMSK | PV | LEV | TBEV | YFV |
| prM | 125-ESWILRNPGYALVA-138 | 5 | 25|524 |  |  |  |  | 100|29 |  |  |
|  | 158-LLLLVAPAYS-167 | 7 |  | 98\|100 |  |  |  |  |  |  |
| E | 104-GCGLFGKGSIDTCA-117 | 31 | 52|1295 | 99|245 | 100|15 |  |  | 100|97 | 98|150 | 77|163 |
|  | 293-LKGTTYGVC-301 | 1 |  | 1\|256 |  |  |  |  |  |  |
|  | 370-ELEPPFGDSYIV-381 | 11 | 40\|1402 | 98\|253 |  |  |  | 94\|77 |  |  |
|  | 417-LGDTAWDFGS-426 | 9 | 84\|1296 | 96\|252 |  |  |  | 100\|77 |  |  |
|  | 449-LFGGMSWITQGL-460 | 5 |  | 99\|244 |  |  |  | 100\|77 |  |  |
| NS1 | 114-GWKAWGKSI-122 | 2 |  | 95|58 |  |  |  |  |  |  |
|  | 195-HSDLSYWIES-204 | 4 |  | 95\|57 |  |  |  | 100\|27 |  |  |
|  | 209-TWKLERAVLGEVKSCTWPETHTLWG-233 | 6 |  | 100\|57 |  |  |  | 100\|27 |  |  |
|  | 276-DFDYCPGTTVT-286 | 4 |  | 2\|58 |  |  |  | 96\|27 |  |  |
|  | 313-CRSCTLPPLR-322 | 6 | 92\|335 | 2\|58 |  |  |  |  |  |  |
|  | 328-GCWYGMEIRP-337 | 10 | 97\|329 | 98\|58 |  |  |  | 100\|27 |  |  |
| NS2a | 4-DMIDPFQLGL-13 | 3 |  | 12|58 |  |  |  |  |  |  |
| NS2b | 12-GLMFAIVGGLAELD-25 | 3 |  | 100|55 |  |  |  |  |  |  |
| NS3 | 1-GGVLWDTPSP-10 | 1 | 32|247 |  |  |  |  |  |  |  |
|  | 145-DVIGLYGNGVIMP-157 | 4 | 9\|258 |  |  |  |  |  |  |  |
|  | 191-VLDLHPGAGKTR-202 | 11 | 40\|255 |  |  |  |  | 100\|26 |  |  |
|  | 235-ALRGLPIRY-243 | 2 | 40\|255 |  |  |  |  |  |  |  |
|  | 256-EIVDVMCHATLTHRLMSPHRVPNYNLF-282 | 25 |  | 100\|53 |  |  |  | 100\|26 | 100\|17 | 5\|22 |
|  | 288-HFTDPASIAARGYI-301 | 12 | 80\|245 | 98\|53 |  |  |  | 100\|26 |  | 100\|22 |
|  | 310-AAAIFMTATPPG-321 | 11 | 100\|245 | 100\|53 |  |  |  | 96\|26 |  |  |
|  | 357-GKTVWFVPSV-366 | 8 | 99\|275 |  |  |  |  |  |  |  |
|  | 408-TTDISEMGANF-418 | 34 | 99\|275 | 100\|53 |  |  |  | 100\|26 | 100\|17 |  |
|  | 451-TAASAAQRRGR-461 | 29 | 72\|273 | 100\|53 |  |  |  | 100\|26 | 94\|17 |  |
|  | 526-LRGEERKNFLE-536 | 2 |  | 2\|53 |  |  |  | 100\|26 |  |  |
|  | 540-TADLPVWLA-548 | 3 |  | 100\|53 |  |  |  |  |  |  |
|  | 563-WCFDGPRTNT-572 | 1 |  | 100\|53 |  |  |  |  |  |  |
| NS4a | 43-ALEELPDALQT-53 | 3 |  | 100|52 |  |  |  |  |  |  |
|  | 115-MIVLIPEPEKQRSQTDNQLA-134 | 10 | 35\|239 | 100\|52 |  |  |  | 100\|26 |  |  |
| NS4b | 138-AQRRTAAGIMKN-149 | 10 | 69|248 | 100|52 |  |  |  | 100|26 |  |  |
|  | 156-VATDVPELER-165 | 3 |  | 100\|52 |  |  |  |  |  |  |
| NS5 | 79-DLGCGRGGWCYYMATQK-95 | 36 | 99|246 | 94|52 |  |  | 88|17 | 100|27 | 100|31 | 100|21 |
|  | 107-GPGHEEPQLVQSYGWNIVTMKS-128 | 6 |  |  |  |  |  | 96\|27 |  |  |
|  | 141-DTLLCDIGES-150 | 13 | 99\|245 | 2\|52 |  |  |  |  |  | 100\|21 |
|  | 208-RNPLSRNSTHEMYWVS-223 | 30 | 99\|244 |  |  |  | 100\|17 | 96\|27 | 100\|17 | 100\|21 |
|  | 235-MTSQVLLGRMEK-246 | 1 |  | 100\|52 |  |  |  |  |  |  |
|  | 259-NLGSGTRAVG-268 | 5 |  | 100\|52 |  |  |  |  |  |  |
|  | 299-NHPYRTWNYHGSY-311 | 5 |  |  |  |  |  | 100\|26 |  |  |
|  | 318-SASSLVNGVVRLLSKPWD-335 | 6 |  | 100\|52 |  |  |  | 100\|26 |  |  |
|  | 340-VTTMAMTDTTPFGQQRVFKEKVDTKAPEP-368 | 30 | 100\|306 | 100\|52 |  |  | 100\|27 | 100\|26 | 65\|17 | 100\|21 |
|  | 375-VLNETTNWLW-384 | 1 |  | 100\|52 |  |  |  |  |  |  |
|  | 404-KVNSNAALGAMFEEQNQW-421 | 6 |  | 92\|52 |  |  |  | 96\|26 |  |  |
|  | 451-TCIYNMMGKREK-462 | 34 | 99\|299 | 98\|52 |  |  | 84\|19 | 96\|26 | 88\|17 | 100\|21 |
|  | 472-GSRAIWFMWLGARFLEFEALGFLNEDHWL-500 | 55 | 99\|304 | 100\|52 |  |  |  | 100\|29 | 100\|18 | 100\|22 |
|  | 504-NSGGGVEGLGLQKLGY-519 | 9 |  | 2\|52 |  |  |  |  |  |  |
|  | 533-YADDTAGWDTRIT-545 | 59 | 100\|300 | 98\|52 |  | 100\|13 | 94\|17 | 100\|27 | 100\|18 | 100\|22 |
|  | 548-DLENEAKVLE-557 | 2 |  | 100\|52 |  |  |  |  |  |  |
|  | 571-IELTYRHKVVKVMRP-585 | 10 |  | 98\|52 |  |  |  | 100\|27 |  |  |
|  | 596-ISREDQRGSGQVVTYALNTFTNL-618 | 61 | 3\|302 | 100\|52 |  | 100\|13 | 94\|17 | 100\|27 | 100\|18 | 100\|22 |
|  | 662-RMAVSGDDCVVKPLDDRFA-680 | 30 | 98\|303 | 100\|52 |  |  | 94\|17 | 100\|27 |  | 95\|22 |
|  | 689-MSKVRKDIQEWKPS-702 | 9 |  | 96\|52 |  |  |  | 96\|27 |  |  |
|  | 704-GWYDWQQVPFCSNHFTEL-721 | 7 | 68\|303 | 98\|52 |  |  |  |  |  |  |
|  | 741-GRARISPGAGWNVRDTACLAKSYAQMW-767 | 9 |  | 98\|53 |  |  |  | 100\|27 |  |  |
|  | 769-LLYFHRRDLRLMANAICSAVP-789 | 45 | 100\|302 | 100\|53 |  |  |  | 96\|28 |  |  |
|  | 792-WVPTGRTTWSIH-803 | 38 |  |  |  |  |  | 100\|27 | 92\|24 |  |

*^a^* The species column indicates the total number of viral species that share the pan-WNV sequence

*^b^* Percentage representation of WNV sequences in other viral species is only shown for species with at least a total of 10 sequences reported at NCBI Entrez protein database. These viral species include: DENV, *Dengue virus type 1, 2, 3 or 4*; JEV, *Japanese encephalitis virus*; LIV, *Louping ill* *virus*; OMSK, *Omsk hemorrhagic fever virus*; PV, *Powassan virus*; LEV, *St. Louis encephalitis virus*; TBEV, *Tick-born encephalitis*; and YFV, *Yellow fever virus*. However, despite having a total of ≥ 10 sequences reported, some of these viruses had less than 10 of the relevant conserved sequence (indicated by cells shaded in grey). Empty cells indicate no match between the pan-WNV sequences and the *Flavivirus*.
