# Supplementary material for: Conservation and Variability of West Nile Virus Proteins
Source: PLoS One. 2009 Apr 29;4(4):e5352. doi: 10.1371/journal.pone.0005352 (PMC2670515; doi:10.1371/journal.pone.0005352)
Supplement: Table S2 — Putative HLA supertype-restricted binding nonamer peptides in pan-WNV sequences, predicted by immunoinformatics algorithms (NetCTL, Multipred (MP), ARB and TEPITOPE (TP)). (0.79 MB DOC) [file pone.0005352.s003.doc]

**Table S2**.

| WNV Protein | Pan-WNV Sequence | HLA Supertype-Restriction of Predicted Nonamer Peptide *^a^* | | | | | | | | | | | | | | | | | | | | |
| --- | --- | --- | --- | --- | --- | --- | --- | --- | --- | --- | --- | --- | --- | --- | --- | --- | --- | --- | --- | --- | --- | --- |
|  |  | Class I | | | | | | | | | | | | | | | | | | | Class II | |
|  |  | NetCTL | | | | | | | | | | | | MULTIPRED | | | ARB | | | | MP | TP |
|  |  | A1 | A2 | A3 | A24 | A26 | B7 | B8 | B27 | B39 | B44 | B58 | B62 | | A2 | A3 | A2 | A3 | B7 | B44 | DR | DR |
| prM | 125-ESWILRNPGYALVA-138 |  | | | | | | | | | | | | | | | | | | | | |
|  | 126-SWILRNPGY-134 | A1 |  |  | A24 | A26 |  |  |  |  |  |  | B62 | |  |  |  |  |  |  |  |  |
|  | 127-WILRNPGYA-135 |  |  |  |  |  |  |  |  |  |  |  |  | |  |  |  |  |  |  | DR |  |
|  | 128-ILRNPGYAL-136 |  | A2 |  |  |  | B7 | B8 |  |  |  |  | B62 | |  |  |  |  |  |  | DR |  |
|  | 129-LRNPGYALV-137 |  |  |  |  |  |  |  | B27 |  |  |  |  | |  |  | A2 |  |  |  | DR |  |
|  | 130-RNPGYALVA-138 |  |  |  |  |  |  |  |  |  |  |  |  | |  |  | A2 |  |  |  |  |  |
|  | 158-LLLLVAPAYS-167 |  | | | | | | | | | | | | | | | | | | | | |
|  | 158-LLLLVAPAY-166 | A1 |  | A3 |  | A26 |  | B8 |  |  |  | B58 | B62 | |  |  |  |  |  |  | DR | DR |
|  | 159-LLLVAPAYS-167 |  |  |  |  |  |  |  |  |  |  |  |  | |  |  | A2 |  |  |  | DR | DR |
| E | 1-FNCLGMSNRDF-11 |  | | | | | | | | | | | | | | | | | | | | |
|  | 1-FNCLGMSNR-9 |  |  |  |  |  |  |  |  |  |  |  |  | |  |  |  | A3 |  |  | DR |  |
|  | 104-GCGLFGKGSIDTCA-117 |  | | | | | | | | | | | | | | | | | | | | |
|  | 107-LFGKGSIDT-115 |  |  |  |  |  |  |  |  |  |  |  |  | |  |  |  |  |  |  | DR |  |
|  | 293-LKGTTYGVC-301 |  | | | | | | | | | | | | | | | | | | | | |
|  | 293-LKGTTYGVC-301 |  |  |  |  |  |  |  |  |  |  |  |  | |  |  |  |  |  |  | DR |  |
|  | 338-SVASLNDLTPVGRLVTVNP-356 |  | | | | | | | | | | | | | | | | | | | | |
|  | 338-SVASLNDLT-346 |  |  |  |  |  |  |  |  |  |  |  |  | |  |  | A2 |  |  |  |  |  |
|  | 340-ASLNDLTPV-348 |  | A2 |  |  |  |  |  |  |  |  |  |  | | A2 |  | A2 |  |  |  |  |  |
|  | 346-TPVGRLVTV-354 |  |  |  |  |  | B7 | B8 |  |  |  |  |  | | A2 |  | A2 |  |  |  |  |  |
|  | 370-ELEPPFGDSYIV-381 |  | | | | | | | | | | | | | | | | | | | | |
|  | 371-LEPPFGDSY-379 | A1 |  |  |  |  |  |  |  |  | B44 |  | B62 | |  |  |  |  |  |  |  |  |
|  | 449-LFGGMSWITQGL-460 |  | | | | | | | | | | | | | | | | | | | | |
|  | 449-LFGGMSWIT-457 |  |  |  |  |  |  |  |  |  |  |  |  | |  |  |  |  |  |  | DR |  |
|  | 450-FGGMSWITQ-458 |  |  |  |  |  |  |  |  |  |  |  |  | |  |  |  |  |  |  |  | DR |
|  | 452-GMSWITQGL-460 |  | A2 |  |  |  |  | B8 |  |  |  |  | B62 | |  |  | A2 |  |  |  |  |  |
| NS1 | 58-RSVSRLEHQMW-68 |  | | | | | | | | | | | | | | | | | | | | |
|  | 59-SVSRLEHQM-67 |  |  |  |  | A26 |  |  |  |  |  | B58 | B62 | |  |  |  |  |  |  |  |  |
|  | 60-VSRLEHQMW-68 |  |  |  |  |  |  |  |  |  |  | B58 |  | |  |  |  |  |  |  |  |  |
|  | 154-EVEDFGFGL-162 |  | | | | | | | | | | | | | | | | | | | | |
|  | 154-EVEDFGFGL-162 | A1 |  |  |  | A26 |  |  |  | B39 |  |  |  | |  |  |  |  |  |  |  |  |
|  | 195-HSDLSYWIES-204 |  | | | | | | | | | | | | | | | | | | | | |
|  | 195-HSDLSYWIE-203 | A1 |  |  |  |  |  |  |  |  |  |  |  | |  |  |  |  |  |  |  |  |
|  | 196-SDLSYWIES-204 |  |  |  |  |  |  |  |  |  |  |  |  | |  |  |  |  |  | B44 |  |  |
|  | 209-TWKLERAVLGEVKSCTWPETHTLWG-233 |  | | | | | | | | | | | | | | | | | | | | |
|  | 209-TWKLERAVL-217 |  |  |  | A24 |  |  | B8 |  | B39 |  |  |  | |  |  |  |  |  |  |  |  |
|  | 210-WKLERAVLG-218 |  |  |  |  |  |  |  |  |  |  |  |  | |  |  |  |  |  |  | DR |  |
|  | 211-KLERAVLGE-219 |  |  |  |  |  |  |  |  |  |  |  |  | | A2 |  |  |  |  |  |  |  |
|  | 212-LERAVLGEV-220 |  |  |  |  |  |  |  |  |  | B44 |  |  | |  |  |  |  |  |  |  |  |
|  | 213-ERAVLGEVK-221 |  |  |  |  |  |  |  | B27 |  |  |  |  | |  |  |  |  |  |  |  |  |
|  | 215-AVLGEVKSC-223 |  |  |  |  |  |  |  |  |  |  |  |  | | A2 |  |  |  |  |  |  |  |
|  | 216-VLGEVKSCT-224 |  |  |  |  |  |  |  |  |  |  |  |  | | A2 |  |  |  |  |  |  |  |
|  | 217-LGEVKSCTW-225 |  |  |  |  |  |  |  |  |  |  | B58 |  | |  |  |  |  |  |  |  |  |
|  | 218-GEVKSCTWP-226 |  |  |  |  |  |  |  |  |  | B44 |  |  | |  |  |  |  |  |  |  |  |
|  | 220-VKSCTWPET-228 |  |  |  |  |  |  |  |  |  |  |  |  | |  |  |  |  |  |  | DR |  |
|  | 223-CTWPETHTL-231 | A1 | A2 |  |  |  |  |  |  | B39 |  | B58 |  | | A2 |  |  |  |  |  |  |  |
|  | 224-TWPETHTLW-232 |  |  |  | A24 |  |  |  |  |  |  | B58 |  | |  |  |  |  |  |  |  |  |
|  | 276-DFDYCPGTTVT-286 |  | | | | | | | | | | | | | | | | | | | | |
|  | 277-FDYCPGTTV-285 |  |  |  |  |  |  |  |  |  |  |  |  | |  |  | A2 |  |  | B44 | DR |  |
|  | 313-CRSCTLPPLR-322 |  | | | | | | | | | | | | | | | | | | | | |
|  | 313-CRSCTLPPL-321 |  |  |  |  |  |  |  | B27 | B39 |  |  |  | |  |  |  |  |  |  |  |  |
|  | 314-RSCTLPPLR-322 |  |  | A3 |  |  |  |  |  |  |  |  |  | |  |  |  | A3 |  |  |  |  |
|  | 328-GCWYGMEIRP-337 |  | | | | | | | | | | | | | | | | | | | | |
|  | 328-GCWYGMEIR-336 |  |  |  |  |  |  |  |  |  |  |  |  | |  |  |  | A3 |  |  |  |  |
| NS2a | 4-DMIDPFQLGL-13 |  | | | | | | | | | | | | | | | | | | | | |
|  | 5-MIDPFQLGL-13 | A1 | A2 |  |  |  |  |  |  | B39 |  | B58 |  | | A2 |  | A2 |  |  |  |  |  |
|  | 69-NSGGDVVHLALMATF-83 |  | | | | | | | | | | | | | | | | | | | | |
|  | 69-NSGGDVVHL-77 |  |  |  |  |  |  |  |  | B39 |  |  |  | |  |  |  |  |  |  |  |  |
|  | 71-GGDVVHLAL-79 |  |  |  |  |  |  |  |  | B39 |  |  |  | |  |  |  |  |  |  |  |  |
|  | 72-GDVVHLALM-80 |  |  |  |  |  |  |  |  |  |  |  |  | |  |  |  |  |  | B44 |  |  |
|  | 74-VVHLALMAT-82 |  |  |  |  |  |  |  |  |  |  |  |  | |  |  |  |  |  |  | DR | DR |
|  | 75-VHLALMATF-83 |  |  |  | A24 |  |  | B8 | B27 | B39 |  |  |  | |  |  |  |  |  |  | DR |  |
| NS2b | 1-GWPATEVMTA-10 |  | | | | | | | | | | | | | | | | | | | | |
|  | 2-WPATEVMTA-10 |  |  |  |  |  | B7 |  |  |  |  |  |  | |  |  |  |  | B7 |  | DR |  |
|  | 12-GLMFAIVGGLAELD-25 |  | | | | | | | | | | | | | | | | | | | | |
|  | 13-LMFAIVGGL-21 |  | A2 |  |  |  |  |  | B27 |  |  |  | B62 | | A2 |  | A2 |  |  |  | DR | DR |
|  | 14-MFAIVGGLA-22 |  |  |  |  |  |  |  |  |  |  |  |  | |  |  |  |  |  |  | DR |  |
|  | 15-FAIVGGLAE-23 |  |  |  |  |  |  |  |  |  |  |  |  | |  |  |  |  |  |  | DR | DR |
|  | 16-AIVGGLAEL-24 |  | A2 |  |  | A26 | B7 |  |  |  |  |  | B62 | | A2 |  | A2 |  |  |  |  |  |
|  | 17-IVGGLAELD-25 |  |  |  |  |  |  |  |  |  |  |  |  | |  |  |  |  |  |  | DR |  |
|  | 32-PMTIAGLMF-40 |  | | | | | | | | | | | | | | | | | | | | |
|  | 32-PMTIAGLMF-40 | A1 |  |  | A24 |  |  |  |  |  |  | B58 | B62 | |  |  |  |  |  |  |  |  |
|  | 108-SAYTPWAILPS-118 |  | | | | | | | | | | | | | | | | | | | | |
|  | 108-SAYTPWAIL-116 |  |  |  |  |  | B7 |  |  | B39 |  | B58 | B62 | |  |  |  |  |  |  |  |  |
|  | 110-YTPWAILPS-118 |  |  |  |  |  |  |  |  |  |  |  |  | |  |  | A2 |  |  |  | DR | DR |
| NS3 | 52-TTKGAALMSG-61 |  | | | | | | | | | | | | | | | | | | | | |
|  | 52-TTKGAALMS-60 |  |  |  |  |  |  |  |  |  |  |  |  | |  | A3 |  |  |  |  |  |  |
|  | 63-GRLDPYWGSV-72 |  | | | | | | | | | | | | | | | | | | | | |
|  | 63-GRLDPYWGS-71 |  |  |  |  |  |  |  | B27 |  |  |  |  | |  |  |  |  |  |  |  |  |
|  | 64-RLDPYWGSV-72 | A1 | A2 |  |  |  |  |  |  |  |  |  |  | |  |  | A2 |  |  |  |  |  |
|  | 74-EDRLCYGGPW-83 |  | | | | | | | | | | | | | | | | | | | | |
|  | 75-DRLCYGGPW-83 |  |  |  |  |  |  |  | B27 |  |  |  |  | |  |  |  |  |  |  |  |  |
|  | 108-NVQTKPGVFKTP-119 |  | | | | | | | | | | | | | | | | | | | | |
|  | 108-NVQTKPGVF-116 | A1 |  |  | A24 |  | B7 | B8 |  |  |  |  | B62 | |  |  |  |  |  |  |  |  |
|  | 109-VQTKPGVFK-117 |  |  | A3 |  |  |  |  |  |  |  |  |  | |  | A3 |  |  |  |  | DR |  |
|  | 110-QTKPGVFKT-118 |  |  |  |  |  |  |  |  |  |  |  |  | |  | A3 |  |  |  |  |  |  |
|  | 131-PTGTSGSPIVDK-142 |  | | | | | | | | | | | | | | | | | | | | |
|  | 134-TSGSPIVDK-142 |  |  | A3 |  |  |  |  |  |  |  |  |  | |  | A3 |  |  |  |  |  |  |
|  | 145-DVIGLYGNGVIMP-157 |  | | | | | | | | | | | | | | | | | | | | |
|  | 146-VIGLYGNGV-154 |  |  |  |  |  |  |  |  |  |  |  |  | |  |  | A2 |  |  |  | DR |  |
|  | 147-IGLYGNGVI-155 |  |  |  |  |  |  |  |  |  |  |  |  | |  |  |  |  |  |  | DR |  |
|  | 148-GLYGNGVIM-156 |  | A2 |  |  |  |  |  |  |  |  |  | B62 | |  |  |  |  |  |  |  |  |
|  | 149-LYGNGVIMP-157 |  |  |  |  |  |  |  |  |  |  |  |  | |  |  |  |  |  |  | DR |  |
|  | 161-YISAIVQGERM-171 |  | | | | | | | | | | | | | | | | | | | | |
|  | 161-YISAIVQGE-169 |  |  |  |  |  |  |  |  |  |  |  |  | | A2 |  |  |  |  |  | DR |  |
|  | 162-ISAIVQGER-170 |  |  | A3 |  |  |  |  |  |  |  |  |  | |  | A3 |  | A3 |  |  | DR |  |
|  | 163-SAIVQGERM-171 |  |  |  |  | A26 |  |  |  |  |  | B58 |  | |  |  |  |  |  |  |  |  |
|  | 235-ALRGLPIRY-243 |  | | | | | | | | | | | | | | | | | | | | |
|  | 235-ALRGLPIRY-243 | A1 |  | A3 |  | A26 |  |  |  |  |  |  | B62 | |  | A3 |  |  |  |  |  |  |
|  | 256-EIVDVMCHATLTHRLMSPHRVPNYNLF-282 |  | | | | | | | | | | | | | | | | | | | | |
|  | 256-EIVDVMCHA-264 |  |  |  |  | A26 |  |  |  |  |  |  |  | | A2 |  |  |  |  |  |  |  |
|  | 257-IVDVMCHAT-265 |  |  |  |  |  |  |  |  |  |  |  |  | |  |  |  |  |  |  | DR |  |
|  | 258-VDVMCHATL-266 |  |  |  |  |  |  | B8 |  |  | B44 |  |  | |  |  |  |  |  | B44 |  |  |
|  | 259-DVMCHATLT-267 |  |  |  |  |  |  |  |  |  |  |  |  | |  | A3 | A2 |  |  |  |  |  |
|  | 260-VMCHATLTH-268 |  |  | A3 |  |  |  |  |  |  |  |  | B62 | | A2 |  |  | A3 |  |  | DR | DR |
|  | 261-MCHATLTHR-269 |  |  | A3 |  |  |  |  |  |  |  |  |  | |  |  | A2 | A3 |  |  | DR |  |
|  | 262-CHATLTHRL-270 |  |  |  |  |  |  |  |  | B39 |  |  |  | |  |  |  |  |  |  |  |  |
|  | 263-HATLTHRLM-271 |  |  |  |  |  | B7 | B8 |  |  |  |  |  | |  |  |  |  |  |  |  |  |
|  | 264-ATLTHRLMS-272 |  |  |  |  |  |  |  |  |  |  |  |  | |  | A3 |  |  |  |  |  |  |
|  | 265-TLTHRLMSP-273 |  |  |  |  |  |  |  |  |  |  |  |  | | A2 |  |  |  |  |  |  |  |
|  | 266-LTHRLMSPH-274 |  |  | A3 |  |  |  |  |  |  |  |  |  | |  |  |  | A3 |  |  | DR |  |
|  | 268-HRLMSPHRV-276 |  |  |  |  |  |  |  | B27 | B39 |  |  |  | | A2 |  |  |  |  |  |  |  |
|  | 270-LMSPHRVPN-278 |  |  |  |  |  |  |  |  |  |  |  |  | |  |  |  |  |  |  | DR |  |
|  | 271-MSPHRVPNY-279 | A1 |  | A3 |  | A26 |  |  |  |  |  | B58 | B62 | |  |  |  |  |  |  |  |  |
|  | 273-PHRVPNYNL-281 |  |  |  |  |  |  |  |  | B39 |  |  |  | |  |  |  |  |  |  |  |  |
|  | 274-HRVPNYNLF-282 | A1 |  |  | A24 |  |  | B8 | B27 | B39 |  |  |  | |  |  |  |  |  |  |  |  |
|  | 288-HFTDPASIAARGYI-301 |  | | | | | | | | | | | | | | | | | | | | |
|  | 289-FTDPASIAA-297 | A1 | A2 |  |  |  |  |  |  |  |  |  |  | |  |  | A2 |  |  |  |  |  |
|  | 292-PASIAARGY-300 | A1 |  |  |  |  |  |  |  |  |  |  |  | |  |  |  |  |  |  |  |  |
|  | 293-ASIAARGYI-301 |  |  |  |  |  |  |  |  |  |  |  |  | |  |  |  | A3 |  |  |  |  |
|  | 310-AAAIFMTATPPG-321 |  | | | | | | | | | | | | | | | | | | | | |
|  | 310-AAAIFMTAT-318 |  |  |  |  |  | B7 |  |  |  |  |  |  | |  |  | A2 |  |  |  |  |  |
|  | 313-IFMTATPPG-321 |  |  |  |  |  |  |  |  |  |  |  |  | |  |  |  |  |  |  | DR | DR |
|  | 337-QTEIPDRAWN-346 |  | | | | | | | | | | | | | | | | | | | | |
|  | 337-QTEIPDRAW-345 |  |  |  |  |  |  |  |  |  |  | B58 |  | |  |  |  |  |  |  |  |  |
|  | 357-GKTVWFVPSV-366 |  | | | | | | | | | | | | | | | | | | | | |
|  | 358-KTVWFVPSV-366 |  | A2 |  |  | A26 |  |  |  |  |  | B58 |  | | A2 |  | A2 |  |  |  |  |  |
|  | 385-QLNRKSYETEYPKCKN-400 |  | | | | | | | | | | | | | | | | | | | | |
|  | 385-QLNRKSYET-393 |  |  |  |  |  |  |  |  |  |  |  |  | | A2 | A3 |  |  |  |  |  |  |
|  | 387-NRKSYETEY-395 | A1 |  |  |  |  |  | B8 | B27 |  |  |  |  | |  |  |  |  |  |  |  |  |
|  | 389-KSYETEYPK-397 |  |  | A3 |  |  |  |  | B27 |  |  |  |  | |  | A3 |  | A3 |  |  |  |  |
|  | 391-YETEYPKCK-399 |  |  |  |  |  |  |  |  |  | B44 |  |  | |  |  |  |  |  |  |  |  |
|  | 408-TTDISEMGANF-418 |  | | | | | | | | | | | | | | | | | | | | |
|  | 408-TTDISEMGA-416 | A1 |  |  |  |  |  |  |  |  |  |  |  | |  |  |  |  |  |  |  |  |
|  | 410-DISEMGANF-418 | A1 |  |  |  | A26 |  |  |  |  |  |  | B62 | |  |  |  |  |  |  |  |  |
|  | 422-RVIDSRKSVKP-432 |  | | | | | | | | | | | | | | | | | | | | |
|  | 422-RVIDSRKSV-430 |  |  |  |  |  | B7 |  |  |  |  |  | B62 | | A2 |  |  |  |  |  |  |  |
|  | 423-VIDSRKSVK-431 |  |  | A3 |  |  |  |  |  |  |  |  |  | |  | A3 |  |  |  |  |  |  |
|  | 451-TAASAAQRRGR-461 |  | | | | | | | | | | | | | | | | | | | | |
|  | 451-TAASAAQRR-459 |  |  | A3 |  |  |  |  |  |  |  |  |  | |  | A3 |  | A3 |  |  |  |  |
|  | 453-ASAAQRRGR-461 |  |  |  |  |  |  |  |  |  |  |  |  | |  | A3 |  | A3 |  |  |  |  |
|  | 526-LRGEERKNFLE-536 |  | | | | | | | | | | | | | | | | | | | | |
|  | 526-LRGEERKNF-534 |  |  |  |  |  |  |  | B27 |  |  |  |  | |  |  |  |  |  |  |  |  |
|  | 527-RGEERKNFL-535 |  |  |  |  |  |  | B8 |  |  |  |  |  | |  |  |  |  |  |  |  |  |
|  | 540-TADLPVWLA-548 |  | | | | | | | | | | | | | | | | | | | | |
|  | 540-TADLPVWLA-548 | A1 |  |  |  |  |  |  |  |  |  |  |  | |  |  | A2 |  |  |  |  |  |
|  | 563-WCFDGPRTNT-572 |  | | | | | | | | | | | | | | | | | | | | |
|  | 563-WCFDGPRTN-571 |  |  |  |  |  |  |  |  |  |  |  |  | |  |  |  |  |  |  | DR |  |
| NS4a | 19-KTWEALDTMYVVATA-33 |  | | | | | | | | | | | | | | | | | | | | |
|  | 19-KTWEALDTM-27 |  | A2 |  |  | A26 |  |  |  |  |  | B58 |  | | A2 |  | A2 |  |  |  |  |  |
|  | 20-TWEALDTMY-28 | A1 |  |  |  |  |  |  |  |  |  |  |  | |  |  |  |  |  |  |  |  |
|  | 21-WEALDTMYV-29 |  |  |  |  |  |  |  |  |  | B44 |  |  | |  |  | A2 |  |  | B44 | DR | DR |
|  | 22-EALDTMYVV-30 |  | A2 |  |  | A26 |  |  |  |  |  |  |  | | A2 |  |  |  |  |  |  |  |
|  | 23-ALDTMYVVA-31 | A1 | A2 |  |  |  |  |  |  |  |  |  |  | | A2 |  | A2 |  |  |  |  |  |
|  | 24-LDTMYVVAT-32 |  |  |  |  |  |  |  |  |  |  |  |  | |  |  |  |  |  | B44 | DR |  |
|  | 25-DTMYVVATA-33 |  |  |  |  | A26 |  |  |  |  |  |  |  | |  |  | A2 |  |  |  |  |  |
|  | 43-ALEELPDALQT-53 |  | | | | | | | | | | | | | | | | | | | | |
|  | 43-ALEELPDAL-51 |  | A2 |  |  |  |  |  |  | B39 |  |  |  | | A2 |  |  |  |  |  |  |  |
|  | 45-EELPDALQT-53 |  |  |  |  |  |  |  |  |  | B44 |  |  | |  |  |  |  |  |  |  |  |
|  | 101-GTKIAGMLLLSLL-113 |  | | | | | | | | | | | | | | | | | | | | |
|  | 101-GTKIAGMLL-109 | A1 |  |  |  |  |  |  |  |  |  |  |  | |  |  |  |  |  |  |  |  |
|  | 102-TKIAGMLLL-110 |  |  |  |  | A26 |  |  |  | B39 |  |  |  | | A2 |  |  |  |  |  |  |  |
|  | 103-KIAGMLLLS-111 |  |  | A3 |  |  |  |  |  |  |  |  |  | | A2 |  | A2 |  |  |  |  |  |
|  | 104-IAGMLLLSL-112 |  | A2 |  |  |  | B7 |  |  |  |  |  |  | |  |  |  |  |  |  | DR | DR |
|  | 115-MIVLIPEPEKQRSQTDNQLA-134 |  | | | | | | | | | | | | | | | | | | | | |
|  | 115-MIVLIPEPE-123 |  |  |  |  |  |  |  |  |  |  |  |  | |  |  |  |  |  |  | DR |  |
|  | 116-IVLIPEPEK-124 |  |  | A3 |  |  |  |  |  |  |  |  |  | |  | A3 |  | A3 |  |  |  |  |
|  | 118-LIPEPEKQR-126 |  |  |  |  |  |  |  |  |  |  |  |  | |  | A3 |  | A3 |  |  |  |  |
|  | 125-QRSQTDNQL-133 |  |  |  |  |  |  |  | B27 | B39 |  |  |  | |  |  |  |  |  |  |  |  |
| NS4b | 68-TSLTSINVQASAL-80 |  | | | | | | | | | | | | | | | | | | | | |
|  | 69-SLTSINVQA-77 |  | A2 |  |  |  |  |  |  |  |  |  |  | | A2 |  | A2 |  |  |  |  |  |
|  | 70-LTSINVQAS-78 |  |  |  |  |  |  |  |  |  |  |  |  | |  |  |  |  |  |  | DR | DR |
|  | 71-TSINVQASA-79 |  |  |  |  | A26 |  |  |  |  |  |  |  | |  |  | A2 |  |  |  |  |  |
|  | 72-SINVQASAL-80 |  |  |  |  |  | B7 | B8 |  |  |  |  | B62 | | A2 |  |  |  |  |  |  |  |
|  | 138-AQRRTAAGIMKN-149 |  | | | | | | | | | | | | | | | | | | | | |
|  | 138-AQRRTAAGI-146 |  |  |  |  |  |  |  |  |  |  |  | B62 | | A2 |  |  |  |  |  |  |  |
|  | 139-QRRTAAGIM-147 |  |  |  |  |  |  |  | B27 |  |  |  |  | |  |  |  |  |  |  |  |  |
|  | 140-RRTAAGIMK-148 |  |  |  |  |  |  |  | B27 |  |  |  |  | |  | A3 |  |  |  |  |  |  |
|  | 141-RTAAGIMKN-149 |  |  | A3 |  |  |  |  |  |  |  |  |  | |  |  |  |  |  |  |  |  |
|  | 156-VATDVPELER-165 |  | | | | | | | | | | | | | | | | | | | | |
|  | 156-VATDVPELE-164 |  |  |  |  |  |  |  |  |  |  |  |  | |  |  |  |  |  |  | DR |  |
|  | 157-ATDVPELER-165 | A1 |  |  |  |  |  |  |  |  |  |  |  | |  | A3 |  | A3 |  |  |  |  |
|  | 208-VTLWENGASSVWNATTAIGLCH-229 |  | | | | | | | | | | | | | | | | | | | | |
|  | 209-TLWENGASS-217 |  |  |  |  |  |  |  |  |  |  |  |  | | A2 |  | A2 |  |  |  |  |  |
|  | 210-LWENGASSV-218 |  |  |  |  |  |  |  |  |  |  |  |  | | A2 |  |  |  |  |  |  |  |
|  | 211-WENGASSVW-219 |  |  |  |  |  |  |  |  |  | B44 | B58 | B62 | |  |  |  |  |  |  |  |  |
|  | 215-ASSVWNATT-223 | A1 |  |  |  |  |  |  |  |  |  |  |  | |  |  |  |  |  |  |  |  |
|  | 217-SVWNATTAI-225 |  | A2 |  |  | A26 | B7 |  |  |  |  |  | B62 | | A2 |  | A2 |  |  |  |  |  |
|  | 219-WNATTAIGL-227 |  |  |  |  |  |  |  |  |  |  |  |  | |  |  | A2 |  |  |  | DR |  |
|  | 221-ATTAIGLCH-229 | A1 |  | A3 |  |  |  |  |  |  |  |  |  | |  | A3 |  | A3 |  |  |  |  |
| NS5 | 60-AKLRWLVER-68 |  | | | | | | | | | | | | | | | | | | | | |
|  | 60-AKLRWLVER-68 |  |  |  |  |  |  |  | B27 |  |  |  |  | |  | A3 |  |  |  |  |  |  |
|  | 79-DLGCGRGGWCYYMATQK-95 |  | | | | | | | | | | | | | | | | | | | | |
|  | 81-GCGRGGWCY-89 | A1 |  |  |  |  |  |  |  |  |  |  |  | |  |  |  |  |  |  |  |  |
|  | 82-CGRGGWCYY-90 | A1 |  |  |  | A26 |  |  |  |  |  |  | B62 | |  |  |  |  |  |  |  |  |
|  | 83-GRGGWCYYM-91 |  |  |  |  |  |  |  | B27 |  |  |  |  | |  |  |  |  |  |  |  |  |
|  | 87-WCYYMATQK-95 |  |  | A3 |  |  |  |  |  |  |  |  |  | |  | A3 |  | A3 |  |  | DR |  |
|  | 107-GPGHEEPQLVQSYGWNIVTMKS-128 |  | | | | | | | | | | | | | | | | | | | | |
|  | 107-GPGHEEPQL-115 |  |  |  |  |  | B7 |  |  |  |  |  |  | |  |  |  |  |  |  |  |  |
|  | 111-EEPQLVQSY-119 |  |  |  |  | A26 |  |  |  |  | B44 |  |  | |  |  |  |  |  |  |  |  |
|  | 115-LVQSYGWNI-123 |  | A2 |  |  |  |  |  |  |  |  |  |  | | A2 |  | A2 |  |  |  | DR |  |
|  | 116-VQSYGWNIV-124 |  |  |  |  |  |  |  |  |  |  |  | B62 | |  |  | A2 |  |  |  | DR |  |
|  | 118-SYGWNIVTM-126 |  |  |  | A24 |  |  |  |  | B39 |  |  |  | |  |  |  |  |  |  |  |  |
|  | 119-YGWNIVTMK-127 |  |  |  |  |  |  |  | B27 |  |  |  |  | |  |  |  |  |  |  |  |  |
|  | 141-DTLLCDIGES-150 |  | | | | | | | | | | | | | | | | | | | | |
|  | 142-TLLCDIGES-150 |  |  |  |  |  |  |  |  |  |  |  |  | |  |  | A2 |  |  |  |  |  |
|  | 152-SSAEVEEHRT-161 |  | | | | | | | | | | | | | | | | | | | | |
|  | 152-SSAEVEEHR-160 |  |  |  |  |  |  |  |  |  |  |  |  | |  | A3 |  | A3 |  |  |  |  |
|  | 168-VEDWLHRGP-176 |  | | | | | | | | | | | | | | | | | | | | |
|  | 168-VEDWLHRGP-176 |  |  |  |  |  |  |  |  |  | B44 |  |  | |  |  |  |  |  |  |  |  |
|  | 208-RNPLSRNSTHEMYWVS-223 |  | | | | | | | | | | | | | | | | | | | | |
|  | 211-LSRNSTHEM-219 |  |  |  |  |  | B7 |  |  |  |  | B58 | B62 | |  |  |  |  | B7 |  | DR |  |
|  | 212-SRNSTHEMY-220 | A1 |  |  |  |  |  | B8 | B27 |  |  |  |  | |  |  |  |  |  |  |  |  |
|  | 213-RNSTHEMYW-221 |  |  |  |  |  |  |  |  |  |  | B58 |  | |  |  |  |  |  |  |  |  |
|  | 235-MTSQVLLGRMEK-246 |  | | | | | | | | | | | | | | | | | | | | |
|  | 235-MTSQVLLGR-243 | A1 |  | A3 |  |  |  |  |  |  |  |  |  | | A2 | A3 | A2 | A3 |  |  |  |  |
|  | 238-QVLLGRMEK-246 |  |  | A3 |  |  |  |  |  |  |  |  |  | |  | A3 |  | A3 |  |  |  |  |
|  | 259-NLGSGTRAVG-268 |  | | | | | | | | | | | | | | | | | | | | |
|  | 259-NLGSGTRAV-267 |  | A2 |  |  |  |  |  |  |  |  |  |  | | A2 |  | A2 |  |  |  |  |  |
|  | 299-NHPYRTWNYHGSY-311 |  | | | | | | | | | | | | | | | | | | | | |
|  | 299-NHPYRTWNY-307 | A1 |  |  |  |  |  |  |  |  |  |  |  | |  |  |  |  |  |  |  |  |
|  | 302-YRTWNYHGS-310 |  |  |  |  |  |  |  | B27 |  |  |  |  | |  |  |  |  |  |  | DR |  |
|  | 303-RTWNYHGSY-311 | A1 |  | A3 |  | A26 |  | B8 | B27 |  |  | B58 | B62 | |  | A3 |  | A3 |  |  |  |  |
|  | 318-SASSLVNGVVRLLSKPWD-335 |  | | | | | | | | | | | | | | | | | | | | |
|  | 318-SASSLVNGV-326 |  | A2 |  |  |  |  |  |  |  |  |  |  | | A2 |  | A2 |  |  |  |  |  |
|  | 319-ASSLVNGVV-327 | A1 |  |  |  |  |  |  |  |  |  |  |  | |  |  |  |  |  |  |  |  |
|  | 320-SSLVNGVVR-328 |  |  |  |  |  |  |  |  |  |  |  |  | |  | A3 |  | A3 |  |  |  |  |
|  | 321-SLVNGVVRL-329 | A1 | A2 |  |  | A26 |  |  |  | B39 |  |  | B62 | | A2 |  | A2 |  |  |  |  |  |
|  | 322-LVNGVVRLL-330 |  | A2 |  |  |  |  |  |  |  |  |  | B62 | | A2 |  | A2 |  |  |  | DR |  |
|  | 323-VNGVVRLLS-331 |  |  |  |  |  |  |  |  |  |  |  |  | |  |  |  |  |  |  | DR | DR |
|  | 326-VVRLLSKPW-334 |  |  |  |  |  | B7 |  |  |  |  |  | B62 | |  |  |  |  |  |  | DR | DR |
|  | 327-VRLLSKPWD-335 |  |  |  |  |  |  |  |  |  |  |  |  | |  |  |  |  |  |  | DR | DR |
|  | 340-VTTMAMTDTTPFGQQRVFKEKVDTKAPEP-368 |  | | | | | | | | | | | | | | | | | | | | |
|  | 340-VTTMAMTDT-348 |  |  |  |  |  |  |  |  |  |  |  |  | |  |  |  |  |  |  | DR |  |
|  | 343-MAMTDTTPF-351 | A1 |  |  | A24 |  | B7 | B8 |  |  |  | B58 | B62 | |  |  |  |  | B7 |  | DR |  |
|  | 345-MTDTTPFGQ-353 | A1 |  |  |  |  |  |  |  |  |  |  |  | |  |  |  |  |  |  |  |  |
|  | 347-DTTPFGQQR-355 |  |  |  |  |  |  |  |  |  |  |  |  | |  | A3 |  | A3 |  |  |  |  |
|  | 348-TTPFGQQRV-356 | A1 |  |  |  | A26 |  |  |  |  |  |  |  | |  |  |  |  |  |  |  |  |
|  | 349-TPFGQQRVF-357 |  |  |  |  |  | B7 | B8 |  |  |  |  | B62 | |  |  |  |  | B7 |  |  |  |
|  | 350-PFGQQRVFK-358 |  |  |  |  |  |  |  |  |  |  |  |  | |  | A3 |  |  |  |  |  |  |
|  | 351-FGQQRVFKE-359 |  |  |  |  |  |  |  |  |  |  |  |  | |  |  |  |  |  |  | DR |  |
|  | 352-GQQRVFKEK-360 |  |  | A3 |  |  |  |  | B27 |  |  |  |  | |  | A3 |  |  |  |  |  |  |
|  | 356-VFKEKVDTK-364 |  |  |  |  |  |  |  |  |  |  |  |  | |  | A3 |  |  |  |  |  |  |
|  | 375-VLNETTNWLW-384 |  | | | | | | | | | | | | | | | | | | | | |
|  | 375-VLNETTNWL-383 |  | A2 |  |  |  |  |  |  | B39 |  |  | B62 | | A2 |  | A2 |  |  |  |  |  |
|  | 376-LNETTNWLW-384 | A1 |  |  |  |  |  |  |  |  |  | B58 |  | |  |  |  |  |  |  |  |  |
|  | 404-KVNSNAALGAMFEEQNQW-421 |  | | | | | | | | | | | | | | | | | | | | |
|  | 404-KVNSNAALG-412 |  |  |  |  |  |  |  |  |  |  |  |  | |  | A3 |  |  |  |  |  |  |
|  | 405-VNSNAALGA-413 |  |  |  |  |  |  |  |  |  |  |  |  | |  |  | A2 |  |  |  | DR | DR |
|  | 406-NSNAALGAM-414 |  |  |  |  | A26 |  |  |  |  |  |  |  | |  |  |  |  |  |  |  |  |
|  | 407-SNAALGAMF-415 |  |  |  |  | A26 |  |  |  |  |  |  | B62 | |  |  |  |  |  |  |  |  |
|  | 410-ALGAMFEEQ-418 |  |  |  |  |  |  |  |  |  |  |  |  | | A2 |  |  |  |  |  |  |  |
|  | 413-AMFEEQNQW-421 |  |  |  |  |  |  |  |  |  |  | B58 | B62 | | A2 | A3 |  |  |  |  |  |  |
|  | 440-EREAHLRGEC-449 |  | | | | | | | | | | | | | | | | | | | | |
|  | 441-REAHLRGEC-449 |  |  |  |  |  |  |  |  |  | B44 |  |  | |  |  |  |  |  |  |  |  |
|  | 451-TCIYNMMGKREK-462 |  | | | | | | | | | | | | | | | | | | | | |
|  | 451-TCIYNMMGK-459 |  |  |  |  | A26 |  |  |  |  |  |  |  | |  | A3 |  | A3 |  |  |  |  |
|  | 452-CIYNMMGKR-460 |  |  | A3 |  |  |  |  |  |  |  |  |  | |  | A3 |  | A3 |  |  |  |  |
|  | 453-IYNMMGKRE-461 |  |  |  |  |  |  |  |  |  |  |  |  | |  |  |  |  |  |  | DR |  |
|  | 454-YNMMGKREK-462 |  |  |  |  |  |  |  |  |  |  |  |  | |  | A3 |  | A3 |  |  | DR | DR |
|  | 472-GSRAIWFMWLGARFLEFEALGFLNEDHWL-500 |  | | | | | | | | | | | | | | | | | | | | |
|  | 472-GSRAIWFMW-480 |  |  |  |  |  |  |  |  |  |  | B58 |  | |  |  |  |  |  |  |  |  |
|  | 473-SRAIWFMWL-481 |  |  |  |  |  |  |  | B27 | B39 |  |  |  | |  |  |  |  |  |  |  |  |
|  | 475-AIWFMWLGA-483 |  | A2 |  |  |  |  |  |  |  |  |  |  | |  |  | A2 |  |  |  |  |  |
|  | 476-IWFMWLGAR-484 |  |  |  |  |  |  |  |  |  |  |  |  | |  |  |  | A3 |  |  | DR |  |
|  | 477-WFMWLGARF-485 |  |  |  | A24 |  |  | B8 |  |  |  |  | B62 | |  |  |  |  |  |  | DR |  |
|  | 478-FMWLGARFL-486 |  | A2 |  |  |  |  | B8 |  |  |  |  | B62 | |  |  | A2 |  |  |  | DR | DR |
|  | 480-WLGARFLEF-488 | A1 |  |  | A24 |  |  | B8 |  |  |  |  | B62 | | A2 |  |  |  |  |  | DR |  |
|  | 483-ARFLEFEAL-491 |  |  |  |  |  |  | B8 | B27 | B39 | B44 |  |  | |  |  |  |  |  |  |  |  |
|  | 485-FLEFEALGF-493 | A1 |  |  |  |  |  |  |  |  |  |  | B62 | |  |  |  |  |  |  | DR |  |
|  | 486-LEFEALGFL-494 |  |  |  |  |  |  |  |  |  | B44 |  |  | |  |  | A2 |  |  |  |  |  |
|  | 488-FEALGFLNE-496 |  |  |  |  |  |  |  |  |  | B44 |  |  | |  |  |  |  |  |  | DR |  |
|  | 490-ALGFLNEDH-498 |  |  |  |  |  |  |  |  |  |  |  |  | |  |  |  | A3 |  |  |  |  |
|  | 491-LGFLNEDHW-499 |  |  |  |  |  |  |  |  |  |  | B58 |  | |  |  |  |  |  |  |  |  |
|  | 492-GFLNEDHWL-500 |  |  |  | A24 |  |  |  |  |  | B44 |  |  | |  |  |  |  |  |  |  |  |
|  | 504-NSGGGVEGLGLQKLGY-519 |  | | | | | | | | | | | | | | | | | | | | |
|  | 504-NSGGGVEGL-512 |  |  |  |  |  |  |  |  | B39 |  |  |  | |  |  |  |  |  |  |  |  |
|  | 508-GVEGLGLQK-516 |  |  | A3 |  |  |  |  |  |  |  |  |  | |  | A3 |  |  |  |  |  |  |
|  | 509-VEGLGLQKL-517 |  |  |  |  |  |  |  |  |  | B44 |  |  | |  |  |  |  |  |  |  |  |
|  | 511-GLGLQKLGY-519 | A1 |  | A3 |  |  |  |  |  |  |  |  | B62 | |  |  |  |  |  |  |  |  |
|  | 533-YADDTAGWDTRIT-545 |  | | | | | | | | | | | | | | | | | | | | |
|  | 533-YADDTAGWD-541 |  |  |  |  |  |  |  |  |  |  |  |  | |  |  |  |  |  |  | DR |  |
|  | 536-DTAGWDTRI-544 | A1 |  |  |  | A26 |  |  |  |  |  |  |  | | A2 |  |  |  |  |  |  |  |
|  | 548-DLENEAKVLE-557 |  | | | | | | | | | | | | | | | | | | | | |
|  | 548-DLENEAKVL-556 |  |  |  |  |  |  |  |  |  |  |  |  | | A2 |  |  |  |  |  |  |  |
|  | 571-IELTYRHKVVKVMRP-585 |  | | | | | | | | | | | | | | | | | | | | |
|  | 571-IELTYRHKV-579 |  |  |  |  |  |  |  |  |  | B44 |  |  | |  |  |  |  |  | B44 | DR |  |
|  | 572-ELTYRHKVV-580 |  |  |  |  |  |  | B8 |  |  |  |  |  | | A2 |  |  |  |  |  |  |  |
|  | 573-LTYRHKVVK-581 |  |  | A3 |  |  |  |  |  |  |  |  |  | |  | A3 |  | A3 |  |  | DR |  |
|  | 574-TYRHKVVKV-582 |  |  |  | A24 |  |  |  |  |  |  |  |  | | A2 |  |  |  |  |  |  |  |
|  | 575-YRHKVVKVM-583 |  |  |  |  |  |  | B8 | B27 | B39 |  |  |  | |  |  |  |  |  |  | DR | DR |
|  | 576-RHKVVKVMR-584 |  |  |  |  |  |  |  |  |  |  |  |  | |  | A3 |  |  |  |  |  |  |
|  | 596-ISREDQRGSGQVVTYALNTFTNL-618 |  | | | | | | | | | | | | | | | | | | | | |
|  | 600-DQRGSGQVV-608 |  |  |  |  |  |  |  |  |  |  |  | B62 | |  |  |  |  |  |  |  |  |
|  | 602-RGSGQVVTY-610 | A1 |  | A3 |  |  |  |  | B27 |  |  | B58 | B62 | |  |  |  |  |  |  |  |  |
|  | 604-SGQVVTYAL-612 |  |  |  |  |  | B7 | B8 |  | B39 |  |  |  | |  |  |  |  |  |  |  |  |
|  | 606-QVVTYALNT-614 |  |  |  |  |  |  |  |  |  |  |  |  | |  | A3 |  |  |  |  |  |  |
|  | 607-VVTYALNTF-615 | A1 |  |  | A24 | A26 | B7 |  |  |  |  | B58 | B62 | |  |  |  |  |  |  | DR |  |
|  | 608-VTYALNTFT-616 |  |  |  |  |  |  |  |  |  |  |  |  | |  |  | A2 |  |  |  | DR |  |
|  | 609-TYALNTFTN-617 |  |  |  | A24 |  |  |  |  |  |  |  |  | |  |  |  |  |  |  |  |  |
|  | 610-YALNTFTNL-618 | A1 | A2 |  | A24 | A26 | B7 | B8 |  | B39 | B44 |  |  | | A2 |  | A2 |  |  |  | DR |  |
|  | 620-VQLVRMMEGEGV-631 |  | | | | | | | | | | | | | | | | | | | | |
|  | 620-VQLVRMMEG-628 |  |  |  |  |  |  |  |  |  |  |  |  | |  |  |  |  |  |  | DR | DR |
|  | 622-LVRMMEGEG-630 |  |  |  |  |  |  |  |  |  |  |  |  | |  |  |  |  |  |  | DR |  |
|  | 623-VRMMEGEGV-631 |  |  |  |  |  |  |  |  |  |  |  |  | |  |  |  |  |  |  | DR | DR |
|  | 662-RMAVSGDDCVVKPLDDRFA-680 |  | | | | | | | | | | | | | | | | | | | | |
|  | 663-MAVSGDDCV-671 |  |  |  |  |  |  |  |  |  |  |  |  | |  |  | A2 |  | B7 |  |  |  |
|  | 664-AVSGDDCVV-672 |  |  |  |  |  |  |  |  |  |  |  |  | | A2 |  | A2 |  |  |  |  |  |
|  | 667-GDDCVVKPL-675 |  |  |  |  |  |  |  |  |  | B44 |  |  | |  |  |  |  |  | B44 |  |  |
|  | 670-CVVKPLDDR-678 |  |  |  |  |  |  |  |  |  |  |  |  | |  | A3 |  |  |  |  |  |  |
|  | 671-VVKPLDDRF-679 |  |  |  |  |  |  |  |  |  |  |  | B62 | |  |  |  |  |  |  |  |  |
|  | 689-MSKVRKDIQEWKPS-702 |  | | | | | | | | | | | | | | | | | | | | |
|  | 691-KVRKDIQEW-699 |  |  |  |  |  |  |  |  |  |  | B58 | B62 | |  |  |  |  |  |  |  |  |
|  | 692-VRKDIQEWK-700 |  |  |  |  |  |  |  | B27 |  |  |  |  | |  | A3 |  |  |  |  | DR |  |
|  | 694-KDIQEWKPS-702 |  |  |  |  |  |  |  |  |  |  |  |  | |  |  |  |  |  | B44 |  |  |
|  | 704-GWYDWQQVPFCSNHFTEL-721 |  | | | | | | | | | | | | | | | | | | | | |
|  | 705-WYDWQQVPF-713 | A1 |  |  | A24 |  |  | B8 |  | B39 |  |  |  | |  |  |  |  |  |  | DR |  |
|  | 708-WQQVPFCSN-716 |  |  |  |  |  |  |  |  |  |  |  |  | |  |  |  |  |  |  | DR |  |
|  | 709-QQVPFCSNH-717 |  |  |  |  |  |  |  |  |  |  |  | B62 | |  |  |  | A3 |  |  |  |  |
|  | 710-QVPFCSNHF-718 | A1 |  |  | A24 | A26 |  |  |  |  |  |  | B62 | |  |  |  |  |  |  |  |  |
|  | 711-VPFCSNHFT-719 |  |  |  |  |  |  |  |  |  |  |  |  | |  |  |  |  | B7 |  | DR |  |
|  | 713-FCSNHFTEL-721 | A1 |  |  |  |  |  | B8 |  | B39 | B44 |  |  | | A2 |  | A2 |  |  |  |  |  |
|  | 741-GRARISPGAGWNVRDTACLAKSYAQMW-767 |  | | | | | | | | | | | | | | | | | | | | |
|  | 741-GRARISPGA-749 |  |  |  |  |  |  |  | B27 |  |  |  |  | |  |  |  |  |  |  |  |  |
|  | 742-RARISPGAG-750 |  |  |  |  |  | B7 |  |  |  |  |  |  | |  |  |  |  |  |  |  |  |
|  | 743-ARISPGAGW-751 |  |  |  |  |  |  |  | B27 |  |  |  |  | |  |  |  |  |  |  |  |  |
|  | 745-ISPGAGWNV-753 | A1 |  |  |  |  |  |  |  |  |  |  |  | |  |  | A2 |  |  |  |  |  |
|  | 751-WNVRDTACL-759 |  |  |  |  |  |  |  |  | B39 |  |  |  | |  |  | A2 |  |  |  | DR |  |
|  | 752-NVRDTACLA-760 |  |  |  |  |  |  |  |  |  |  |  |  | | A2 |  | A2 |  |  |  |  |  |
|  | 753-VRDTACLAK-761 | A1 |  |  |  |  |  |  | B27 |  |  |  |  | |  |  |  |  |  |  |  |  |
|  | 754-RDTACLAKS-762 |  |  |  |  |  |  |  |  |  |  |  |  | |  |  |  |  |  | B44 |  |  |
|  | 755-DTACLAKSY-763 | A1 |  |  |  | A26 |  |  |  |  |  |  | B62 | |  |  |  |  |  |  |  |  |
|  | 758-CLAKSYAQM-766 |  | A2 |  |  | A26 |  | B8 |  |  |  |  |  | | A2 |  |  |  |  |  |  |  |
|  | 759-LAKSYAQMW-767 |  |  |  |  |  |  |  |  |  |  | B58 |  | |  |  |  |  |  |  | DR |  |
|  | 769-LLYFHRRDLRLMANAICSAVP-789 |  | | | | | | | | | | | | | | | | | | | | |
|  | 769-LLYFHRRDL-777 |  |  |  |  |  |  | B8 |  |  |  |  |  | |  |  |  |  |  |  | DR | DR |
|  | 770-LYFHRRDLR-778 |  |  |  |  |  |  |  |  |  |  |  |  | |  | A3 |  |  |  |  | DR |  |
|  | 771-YFHRRDLRL-779 |  |  |  | A24 |  |  | B8 |  | B39 |  |  |  | | A2 |  |  |  |  |  | DR |  |
|  | 772-FHRRDLRLM-780 |  |  |  |  |  |  | B8 |  |  |  |  |  | |  |  |  |  |  |  | DR |  |
|  | 773-HRRDLRLMA-781 |  |  |  |  |  |  |  | B27 |  |  |  |  | |  |  |  |  |  |  |  |  |
|  | 774-RRDLRLMAN-782 |  |  |  |  |  |  |  | B27 |  |  |  |  | |  |  |  |  |  |  |  |  |
|  | 776-DLRLMANAI-784 |  |  |  |  |  |  | B8 |  |  |  |  |  | | A2 |  |  |  |  |  |  |  |
|  | 777-LRLMANAIC-785 |  |  |  |  |  |  |  |  |  |  |  |  | |  |  |  |  |  |  | DR | DR |
|  | 778-RLMANAICS-786 |  |  | A3 |  |  |  |  |  |  |  |  |  | | A2 | A3 |  |  |  |  |  |  |
|  | 779-LMANAICSA-787 |  | A2 |  |  |  |  |  |  |  |  |  | B62 | | A2 |  | A2 |  |  |  | DR | DR |
|  | 780-MANAICSAV-788 |  | A2 |  |  |  |  |  |  |  |  |  |  | | A2 |  | A2 |  |  |  |  |  |
|  | 792-WVPTGRTTWSIH-803 |  | | | | | | | | | | | | | | | | | | | | |
|  | 792-WVPTGRTTW-800 |  |  |  |  |  | B7 |  |  |  |  | B58 | B62 | |  |  |  |  |  |  |  |  |
|  | 793-VPTGRTTWS-801 |  |  |  |  |  | B7 |  |  |  |  |  |  | |  |  |  |  |  |  |  |  |
|  | 794-PTGRTTWSI-802 |  |  |  | A24 |  |  |  |  |  |  |  |  | |  |  |  |  |  |  |  |  |

^a^ Putative supertypes-restrictions of nonamer sequences with concurring predictions from at least two prediction tools are highlighted in grey. Hotspots with at least three sequential nonamers overlapping by eight amino acids, found in 7 of the 78 pan-WNV sequences, are each indicated by a box.
